# Plasma metabolome mediates the causal relationship between immune cells and heart failure: a two-step bidirectional Mendelian randomization study

**DOI:** 10.3389/fcvm.2024.1430477

**Published:** 2024-10-09

**Authors:** Tan Li, Yanwei Liu, Juncong Fu, Langlang Huang, Zhongyong Liu

**Affiliations:** ^1^ Department of Postgraduate, Jiangxi University of Chinese Medicine, Nanchang, Jiangxi, China; ^2^ Department of Cardiology, Affiliated Hospital of Jiangxi University of Chinese Medicine, Nanchang, Jiangxi, China; ^3^ Department of Traditional Chinese Medicine, Shangrao Municipal Hospital, Shangrao, Jiangxi, China; ^4^ National Pharmaceutical Engineering Center, Jiangxi University of Chinese Medicine, Nanchang, Jiangxi, China

**Keywords:** plasma metabolome, immune cells, heart failure, Mendelian randomization, mediate

## Abstract

**Background:**

Prior research has established a correlation between immune cell activity and heart failure (HF), but the causal nature of this relationship remains unclear. Furthermore, the potential influence of metabolite levels on this interaction has not been comprehensively explored. To address these gaps, we employed a bidirectional Mendelian randomization (MR) approach in two stages to examine whether metabolite levels can mediate the causal relationship between immune cells and HF.

**Methods:**

Genetic information was extracted from summary data of genome-wide association studies. By applying a two-sample, two-step MR approach, we investigated the causal relationships among immune cells, metabolite levels, and HF, with a specific focus on the mediating effects of metabolites. Sensitivity analysis techniques were implemented to ensure the robustness of our findings.

**Results:**

MR analysis revealed significant causal associations between HF and eight specific immune cells and five metabolites. Mediation analysis further identified three mediated relationships. Particularly, hexadecenedioate (C16:1-DC) mediated the influence of both the CD28- CD127- CD25++ CD8br%CD8br (mediation proportion: 19.2%) and CD28+ CD45RA + CD8br%T cells (mediation proportion: 11.9%) on HF. Additionally, the relationship between IgD + CD38br AC cells and HF appeared to be mediated by the phosphate to alanine ratio (mediation proportion: 16.3%). Sensitivity analyses validated that the used instrumental variables were free from pleiotropy and heterogeneity.

**Conclusion:**

This study provides evidence that certain immune cell levels are associated with the risk of HF and that metabolite levels may mediate these relationships. However, to strengthen these findings, further validation using MR analyses with larger sample sizes is essential.

## 1 Introduction

Heart failure (HF) is a clinical syndrome resulting from impaired cardiac structure or function, representing the severe and final stages of various heart diseases. It carries a high prevalence, mortality, and significant economic burden, making it one of the foremost cardiovascular conditions of the 21st century (1). Studies in epidemiology have revealed that there are currently 64.3 million individuals with HF worldwide, with approximately 3 million new cases annually (2). In China, over 12.1 million individuals aged 25 and older are affected by HF (3). A meta-analysis of 1.5 million patients with chronic HF revealed survival rates of 87%, 73%, 57%, and 35% at 1, 2, 5, and 10 years, respectively (4). HF severely affects the health and quality of life of patients, positioning it as a global priority in chronic disease prevention and research.

As the understanding of the pathophysiological mechanisms underlying HF advances, there is growing evidence supporting the involvement of immune activation in the disease development (5, 6). Both innate (monocytes and macrophages) and adaptive (T lymphocytes) immune responses can intensify HF progression by releasing pro-inflammatory factors. Macrophages, the predominant immune cells in the myocardium, are integral in mediating inflammatory responses, maintaining cardiac stability, and promoting tissue repair (7). During the initial stages of HF, macrophages can polarize into the M1 phenotype, exacerbating myocardial damage through the secretion of pro-inflammatory cytokines and chemokines (8). Furthermore, various triggers associated with cardiac injury can activate effector T cells, which infiltrate the vascular walls. The diverse cytokines released by different T cell subsets can then accelerate vascular aging, degrade the elastic lamina, and promote myocardial fibrosis, ultimately altering cardiovascular structure and function (9).

Emerging in the post-genomic era, metabolomics offers a novel perspective by examining the links between metabolites or metabolic pathways and physiological as well as pathological changes, thereby providing new insights into disease mechanisms (10). Compelling evidence indicates that metabolites and metabolic pathways are intricately linked with HF. For example, a targeted serum metabolomic study has identified several metabolites, including octadecanoic acid, tyrosine, and catecholamines, which are significantly associated with HF severity (11). Additionally, metabolites found in plasma, such as ketone bodies and branched-chain amino acids, might play a mediating role in the interaction between immune cells and HF (12, 13).

Although prior research has established links among immune cells, metabolomics, and HF, the precise causal relationships and the mediating effects of plasma metabolites have yet to be clarified. Utilizing Mendel's Second Law, or the Law of Independent Assortment, Mendelian randomization (MR) is an innovative genetic statistical technique that uses genetic variants linked to exposure factors as instrumental variables (IVs) for determining causal links between exposures and outcomes. This method, which relies on the random allocation determined by DNA genotypes, significantly reduces external influences on these causal relationships (14, 15). Mediation analysis further aids in evaluating how exposure affects outcomes through mediators (16). Thus, utilizing publicly available summary data from genome-wide association studies (GWAS), we executed MR analysis to evaluate the causal relationships among immune cells, plasma metabolites, and HF and to elucidate the mediating role of plasma metabolites.

## 2 Research methods and materials

### 2.1 Study design

**Figure 1:**
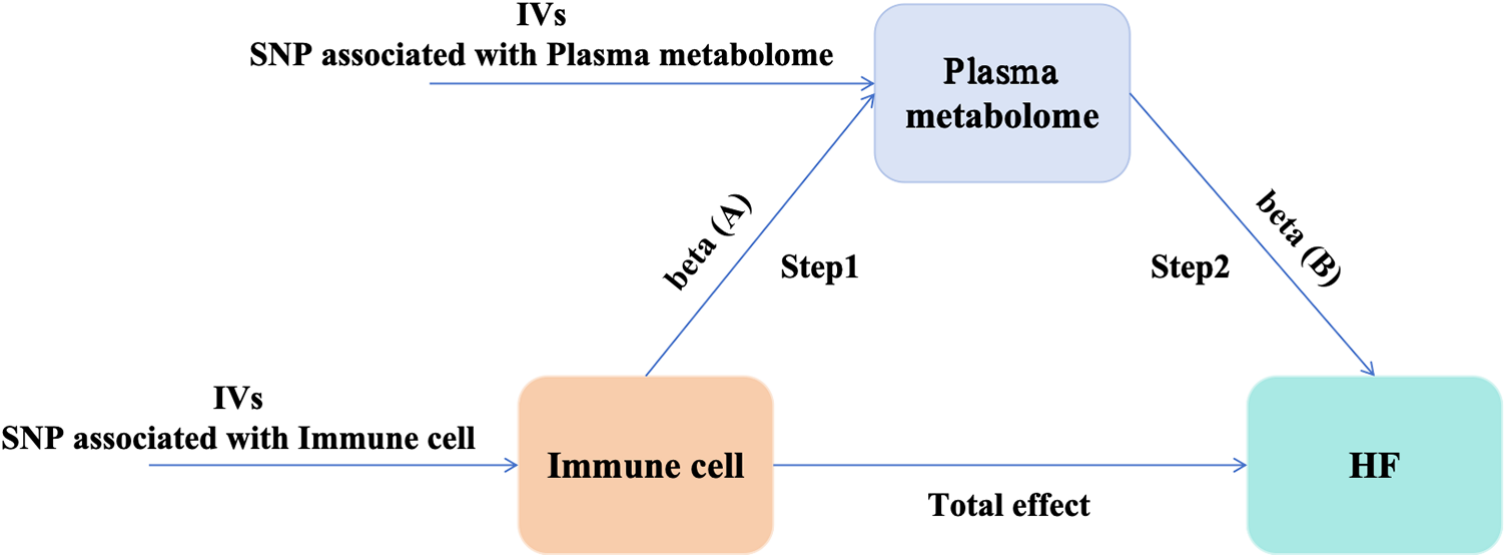
Study design: a two-step MR study of immune cells on HF mediated by plasma metabolome. HF, heart failure; IVs, instrumental variables.

Initially, we accessed genome-wide data on immune cells, plasma metabolites, and HF from publicly available GWAS summary datasets. A two-sample MR analysis was conducted to investigate the causal interactions among immune cells, plasma metabolites, and HF. Subsequently, a two-step MR analysis was undertaken to determine the mediating influence of plasma metabolites on the interaction between immune cells and HF. Figure 1 depicts the schematic representation of the overall design of our study.

### 2.2 Data sources

Summary statistics for immune cells were extracted from the GWAS summary dataset hosted at https://www.ebi.ac.uk/gwas/, encompassing study access numbers GCST90001391 to GCST90002121. This dataset included data on 731 immune phenotypes derived from 3,757 European individuals, covering a wide range of cell types such as T, B, and natural killer cells (TBNK), B cells, CDC, Treg, monocytes, various T cell maturation stages, and a bone marrow cell panel, including absolute cell counts (118), relative counts (192), median fluorescence intensities of surface antigens (389), and morphological parameters (32) (17, 18).

For plasma metabolomics, summary statistics were sourced from the same database, with study access numbers GCST90199621 to GCST90201020. This dataset comprised 1,091 plasma metabolites and 309 metabolite ratios from 8,299 European individuals, spanning categories such as 395 types of lipids, 210 types of amino acids, 22 types of carbohydrates, 33 types of nucleotides, 31 types of vitamins, 21 types of peptides, 8 types of energies, 21 types of characteristic molecules, and 220 types of unknown metabolites, etc. (19).

HF GWAS data were obtained from https://gwas.mrcieu.ac.uk/, under the data ID “ebi-a-GCST009541,” involving a cohort of 977,323 participants, which includes 47,309 HF patients and 930,014 controls. HF GWAS data from 26 cohort studies, with cases including subjects clinically diagnosed with HF of any etiology (20).

### 2.3 Selection of IVs

The selection of IVs must adhere to three core assumptions (16): (1) Single nucleotide polymorphisms (SNPs) utilized as IVs should exhibit a strong association with the exposure factor; (2) SNPs used as IVs must be independent of any confounding factors; (3) SNPs should solely influence the outcome through the exposure factor. To ensure the accuracy and efficacy of the causal links among immune cells, plasma metabolites, and HF risk, we used a genome-wide significance threshold for exposure-related SNPs of *P* < 1 × 10^−5^, following guidelines from earlier MR studies (21, 22). To circumvent bias from linkage disequilibrium within SNPs, we used the PLINK clumping method (*r*^2^ < 0.001, kb = 10,000) based on data from the 1,000 Genomes Project for Europeans to isolate independent SNPs. We then calculated the F-statistics for each SNP, discarding those with *F* < 10 to prevent weak IVs from influencing our results.

### 2.4 MR analysis

#### 2.4.1 Primary analysis

To evaluate the causal impact of immune cells and plasma metabolites on HF, we implemented several MR techniques, encompassing the inverse variance weighted (IVW), MR-Egger regression, weighted median, weighted mode, and simple mode methods. The IVW method is widely acknowledged for its robustness in MR studies for estimating the causal effects of exposure factors on outcomes (23). Consequently, IVW served as the primary analytical tool in our research. The additional methodologies—MR-Egger regression, weighted median, weighted mode, and simple mode—were used to corroborate the primary findings and evaluate the stability of the results.

#### 2.4.2 Mediation analysis

We used a two-step MR approach for mediation analysis to investigate whether plasma metabolites serve as mediators in the disease pathway between immune cells and HF. The total effect was parsed into direct effects (the influence of immune cells on HF) and mediating effects. The causal relationship between immune cells and plasma metabolites was evaluated using the two-sample MR approach, yielding β (A); β (B) was derived from plasma metabolites causally linked to HF. The mediation analysis employed the following formula: Mediating Effect = β (A) × β (B), with the Mediation Proportion calculated as (Mediating Effect/Total Effect) × 100%. The delta method was used to determine the 95% confidence intervals (CI) for both the mediating effect and the mediation proportion.

### 2.5 Sensitivity analysis

We conducted sensitivity analyses using three distinct approaches, namely the MR-Egger intercept, Cochran's Q test, and the leave-one-out method, to determine the potential effects of heterogeneity and horizontal pleiotropy on our findings. Heterogeneity among the SNPs was assessed using Cochran's Q test, where *P* < 0.05 was considered indicative of significant heterogeneity. Horizontal pleiotropy was globally evaluated using the MR-Egger intercept, with *P* < 0.05 suggesting the presence of this effect. Additionally, the leave-one-out method involved sequentially excluding each SNP to observe any resultant variations in the analysis, thereby assessing the robustness of our data.

### 2.6 Statistical analysis

All MR analyses were conducted using R software (version 4.3.1). For estimating causal effects and identifying outliers, we used the “TwoSampleMR” (version 0.5.8) and “MR-PRESSO” (version 1.0) packages. The results are presented as odds ratios (OR) with 95% CI for each standard deviation. Statistical significance for the MR outcomes was set at *P* < 0.05. Finally, multiple testing correction was used to eliminate the increase in Type I error caused by multiple testing, where a resultant false discovery rate (FDR) <0.05 was considered causally related; FDR >0.05 but a *P* value < 0.05 suggests a potential causal relationship; and a *P* value > 0.05 was considered no causal relationship. Using the online power calculation tool (mRnd) (https://cnsgenomics.com/shiny/mRnd/) to calculate the statistical power of the causal effect estimation (24).

## 3 Results

### 3.1 MR analysis of immune cells and HF

**Figure 2:**
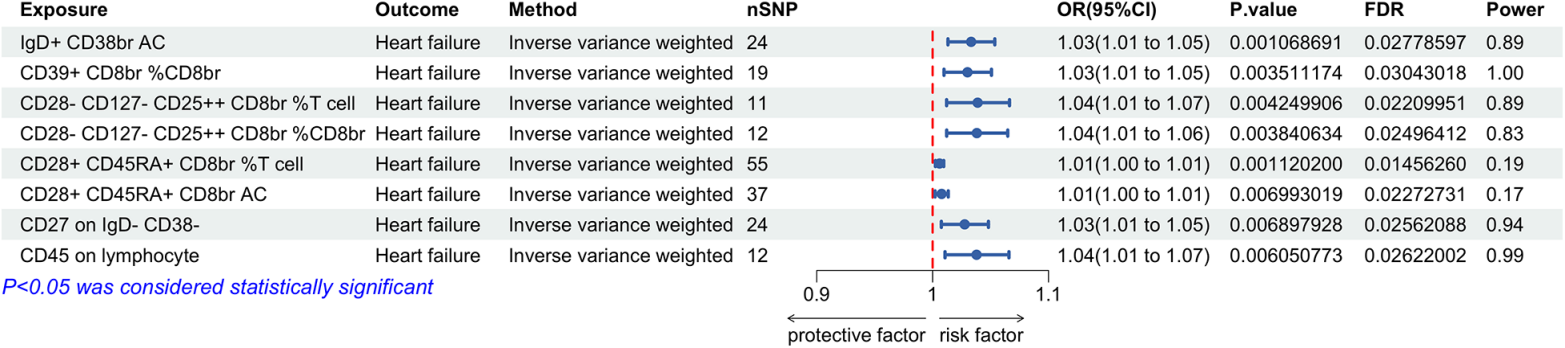
Forest plot for the Mendelian randomization analysis between immune cells and heart failure.

Our analysis revealed that among 731 immune cell phenotypes, 8 demonstrate a significant causal relationship with HF (*P* < 0.01). The IVW results for these immune cells are as follows: IgD + CD38br AC [*P* = 0.001; FDR = 0.028; OR 95% CI = 1.03 (1.01, 1.05)], CD39+ CD8br%CD8br [*P* = 0.004; FDR = 0.030; OR 95% CI = 1.03 (1.01, 1.05)], CD28- CD127- CD25++ CD8br% T cell [*P* = 0.004; FDR = 0.022; OR 95% CI = 1.04 (1.01, 1.07)], CD28- CD127- CD25++ CD8br%CD8br [*P* = 0.004; FDR = 0.025; OR 95% CI = 1.04 (1.01, 1.06)], CD28+ CD45RA + CD8br% T cell [*P* = 0.001; FDR = 0.015; OR 95% CI = 1.01 (1.00, 1.01)], and CD28+ CD45RA + CD8br AC [*P* = 0.007; FDR = 0.023; OR 95% CI = 1.01 (1.00, 1.01)], CD27 on IgD- CD38- [*P* = 0.007; FDR = 0.026; OR 95% CI = 1.03 (1.01, 1.05)], and CD45 on lymphocytes [*P* = 0.006; FDR = 0.026; OR 95% CI = 1.04 (1.01, 1.07)]. Each phenotype showed a positive correlation with HF risk (Figure 2). Sensitivity analyses using the MR-Egger intercept and Cochran's Q test yielded *P*-values greater than 0.05, suggesting no evidence of horizontal pleiotropy or heterogeneity among the IVs. The leave-one-out analysis indicated no significant outliers, validating the stability and reliability of the MR findings. Detailed results of the MR analysis of immune cells and HF are available in Supplementary Tables S3, S4 and Supplementary Figures 1–3.

### 3.2 MR analysis of plasma metabolites and HF

**Figure 3:**
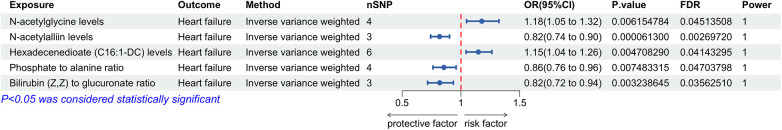
Forest plot for the Mendelian randomization analysis between plasma metabolites and heart failure.

The findings indicated that among 1,400 plasma metabolites, 5 exhibited a significant causal relationship with HF (*P* < 0.01). The IVW results for these metabolites were as follows: N-acetylglycine levels [*P* = 0.006; FDR = 0.045; OR 95% CI = 1.18 (1.05, 1.32)], N-acetylalliin levels [*P* < 0.001; FDR = 0.003; OR 95% CI = 0.82 (0.74, 0.90)], hexadecenedioate (C16:1-DC) levels [*P* = 0.005; FDR = 0.041; OR 95% CI = 1.15 (1.04, 1.26)], phosphate to alanine ratio [*P* = 0.007; FDR = 0.047; OR 95% CI = 0.86 (0.76, 0.96)], and bilirubin (Z,Z) to glucuronate ratio [*P* = 0.003; FDR = 0.036; OR 95% CI = 0.82 (0.72, 0.94)]. N-acetylglycine and hexadecenedioate (C16:1-DC) levels positively correlated with HF risk, whereas N-acetylalliin levels, phosphate to alanine ratio, and bilirubin (Z,Z) to glucuronate ratio negatively correlated with HF risk (Figure 3). The MR-Egger intercept and Cochran's Q tests revealed no significant horizontal pleiotropy or heterogeneity. Leave-one-out analysis showed no biased SNPs, indicating robust results. A detailed discussion of the MR analysis of plasma metabolites and HF is provided in Supplementary Tables S5, S6 and Supplementary Figures S4–S6.

### 3.3 Mediation analysis results

**Figure 4:**
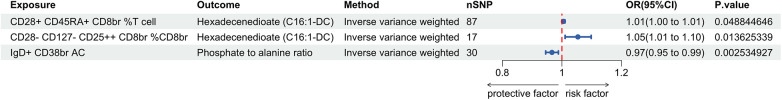
Forest plot for the Mendelian randomization analysis between immune cells and plasma metabolites.

To explore the potential mechanisms driving the onset and progression of HF, we conducted a mediation analysis to identify the causal pathways mediated by plasma metabolites from immune cells to HF. Initially, the causal relationships between immune cells and plasma metabolites were evaluated using two-sample MR. The IVW results showed positive correlations of CD28+ CD45RA + CD8br%T [*P* = 0.049; OR 95% CI = 1.01 (1.00, 1.01)] and CD28- CD127- CD25++ CD8br%CD8br cells [*P* = 0.014; OR 95% CI = 1.05 (1.01, 1.10)] with hexadecenedioate (C16:1-DC) levels. In contrast, IgD + CD38br AC cells [*P* = 0.003; OR 95% CI = 0.97 (0.95, 0.99)] were negatively correlated with the phosphate to alanine ratio (Figure 4). Subsequent mediation analysis revealed that hexadecenedioate (C16:1-DC) mediated the pathways from CD28+ CD45RA + CD8br%T and CD28- CD127- CD25++ CD8br%CD8br cells to HF, with mediation proportions of 11.9% and 19.2%, respectively. Additionally, the phosphate to alanine ratio was found to mediate the link between IgD + CD38br AC cells and HF, with a mediation proportion of 16.3% (Table 1). Further details are provided in Supplementary Table S7 and Supplementary Figures S7–S9.

**Table 1:** Mediation effect of immune cells on heart failure via plasma metabolites.

Exposure	Mediator	Outcome	Mediated effect (95% CI)	*P*-value	Mediated proportion (95% CI)
CD28 + CD45RA + CD8br%T cell	Hexadecenedioate (C16:1-DC)	Heart failure	0.000707 (3.08e-06, 0.00141)	0.049	11.9% (0.0518%, 23.7%)
CD28-CD127- CD25++CD8br%CD8br	Hexadecenedioate (C16:1-DC)	Heart failure	0.00721 (0.00108, 0.0133)	0.021	19.2% (2.89%, 35.6%)
IgD + CD38br AC	Phosphate to alanine ratio	Heart failure	0.00533 (0.00179, 0.00887)	0.003	16.3% (5.47%, 27.1%)

## 4 Discussion

In this study, we utilized a two-sample MR analysis to investigate the causal relationships among immune cells, plasma metabolites, and HF. Our findings demonstrate that IgD + CD38br AC, CD39+ CD8br%CD8br, CD28- CD127- CD25++ CD8br%T, CD28- CD127- CD25++ CD8br%CD8br, CD28+ CD45RA + CD8br%T, and CD28+ CD45RA + CD8br AC cells, CD27 on IgD- CD38- cells, and CD45 on lymphocytes contribute to an increased risk of HF. Similarly, elevated levels of N-acetylglycine and hexadecenedioate (C16:1-DC) are associated with a higher risk of HF, whereas lower risks are linked to N-acetylalliin levels, phosphate to alanine ratio, and bilirubin (Z,Z) to glucuronate ratio. Further analysis reveals that the increased HF risk associated with CD28+ CD45RA + CD8br%T and CD28- CD127- CD25++ CD8br%CD8br cells is mediated by hexadecenedioate (C16:1-DC). Moreover, the heightened HF risk linked to IgD + CD38br AC cells is mediated by the phosphate to alanine ratio.

CD28+ CD45RA + CD8br%T and CD28- CD127- CD25++ CD8br%CD8br cells are classified under the Treg panel, whereas IgD + CD38br AC cells fall under the B cell panel. T lymphocytes, the pivotal immune cells in the development and progression of HF, are categorized into CD4 + and CD8 + subgroups based on surface markers. Once activated, CD4 + cells can differentiate into regulatory and effector T cell subgroups, which include Th1, Th2, Th17, and Tregs (25). Tregs, comprising approximately 5%–10% of all peripheral CD4+ T cells, play a pivotal role in maintaining internal equilibrium, immune homeostasis, and peripheral immune tolerance through the production of anti-inflammatory cytokines such as transforming growth factor β and interleukin 10. These cytokines inhibit the activity of other immune cells, including antigen-presenting cells like macrophages and CD8 + effector T cells, thereby reducing inflammation and preventing hyperactive immune responses (26, 27). Emerging research suggests that a decrease in circulating Treg numbers is associated with an increased risk of cardiovascular diseases. Moreover, Treg counts may serve as valuable biomarkers for predicting exacerbations of HF and the likelihood of rehospitalization (28, 29). In the early stages of cardiac injury, Tregs can mitigate inflammatory responses, generate repair-associated molecules, and directly facilitate repair. However, as HF progresses, Tregs may alter their phenotype and functionality, exacerbating HF (30, 31). CD28, a co-stimulatory molecule expressed on T lymphocytes, is crucial for T cell activation. Lack of CD28 can diminish systemic and cardiac inflammation, suppress T cell activation, and slow the progression of HF (32). In contrast, B cells produce pro-inflammatory factors early in the injury process, recruit monocytes to the heart, and aggravate acute cardiac injury (31). CD38 is a type II transmembrane glycoprotein vital for maintaining intracellular NAD levels. Evidence suggests that a deficiency in CD38 significantly boosts intracellular nicotinamide adenine dinucleotide (NAD) levels across various tissues, curtails oxidative stress pathways, and ameliorates cardiac hypertrophy and myocardial fibrosis (33).

Hexadecenedioate (C16:1-DC), as a lipid, plays a pivotal role in fatty acid metabolism (34). Research indicates that elevated plasma free fatty acid levels can enhance myocardial free fatty acid uptake, thereby augmenting intramyocardial lipid storage and contributing to left ventricular dysfunction (35). Alanine, classified as a non-essential amino acid, is predominantly synthesized through glycolysis and other metabolic pathways (36). Metabolomic studies have demonstrated reductions in alanine levels in the serum of HF models induced by oxidative stress (37). Strong evidence indicates that alterations in energy metabolism exacerbate the severity of HF. Stimulating glucose metabolism or inhibiting fatty acid oxidation can alleviate the effects of decreased mitochondrial oxidative capacity and improve cardiac function (38). Phosphate, a constituent of phospholipids, is crucial for cellular energy metabolism and participates in glycolysis, ammoniagenesis, and oxidative phosphorylation. Persistently high levels of plasma phosphate can elicit inflammatory responses and vascularization, thereby increasing cardiovascular disease morbidity and mortality (39, 40). Thus, for HF patients, monitoring hexadecenedioate (C16:1-DC), alanine, and phosphate levels can aid in disease prevention and early diagnosis, reducing the risk of developing HF. Furthermore, previous research has shown that cellular metabolism regulates immune cell function and differentiation, influencing outcomes in adaptive and innate immune responses. Effector T and Th17 cells rely on aerobic glycolysis, whereas memory T cells and Tregs primarily depend on fatty acid oxidation for energy (41). Our findings affirm the mediating roles of hexadecenedioate (C16:1-DC) and the phosphate to alanine ratio in the associations of CD28+ CD45RA + CD8br%T, CD28- CD127- CD25++ CD8br%CD8br, and IgD + CD38br AC cells with HF. Our mediation analysis indicated that hexadecenedioate (C16:1-DC) contributed to 11.9% and 19.2% of the mediation in the effects of CD28+ CD45RA + CD8br%T and CD28- CD127- CD25++ CD8br%CD8br cells on HF, respectively. The phosphate to alanine ratio mediated 16.3% of the effect of IgD + CD38br AC cells on HF.

Our research boasts several strengths. First, utilizing a two-sample, two-step MR analysis, we examined the causal links and mediating roles of plasma metabolites between immune cells and HF. Second, our study relied on a comprehensive population genetic database, enhancing the reliability of our findings. Additionally, extensive sensitivity analyses were conducted to bolster the robustness of our MR outcomes. Despite these strengths, our study has certain limitations. The data originates from a European cohort, and there may be interactions between diet, genes, and the environment (42), which could constrain the relevance of our findings to other demographic groups. Furthermore, the impact of age varies across the exposure and outcome variables studied. Although HF demonstrates a stronger correlation with age, our data lacks age-specific screening. Future research obtaining age-specific GWAS data could further refine these results through more targeted MR analyses.

## 5 Conclusion

Our MR study has established causal connections between eight specific immune cells, five metabolites, and HF. Additionally, findings from mediation analysis suggest that hexadecenedioate (C16:1-DC) influences HF regulation through CD28+ CD45RA + CD8br%T and CD28- CD127- CD25++ CD8br%CD8br cells. Similarly, the phosphate to alanine ratio affects HF regulation through IgD + CD38br AC cells. These identified immune cells and plasma metabolites can act as valuable biomarkers for diagnosing and treating HF and help in understanding its pathophysiological mechanisms.

## Data Availability

The original contributions presented in the study are included in the article/Supplementary Material, further inquiries can be directed to the corresponding authors.
