# Supplementary material for: Plasma metabolome mediates the causal relationship between immune cells and heart failure: a two-step bidirectional Mendelian randomization study
Source: Front Cardiovasc Med. 2024 Oct 9;11:1430477. doi: 10.3389/fcvm.2024.1430477 (PMC11496177; doi:10.3389/fcvm.2024.1430477)
Supplement: Supplementary file 1 [file Datasheet1.docx]

Supplementary Material

**Supplementary Figure S1.** The scatter plot in MR studies of Immune cells to Heart failure (*P*<0.01).

| 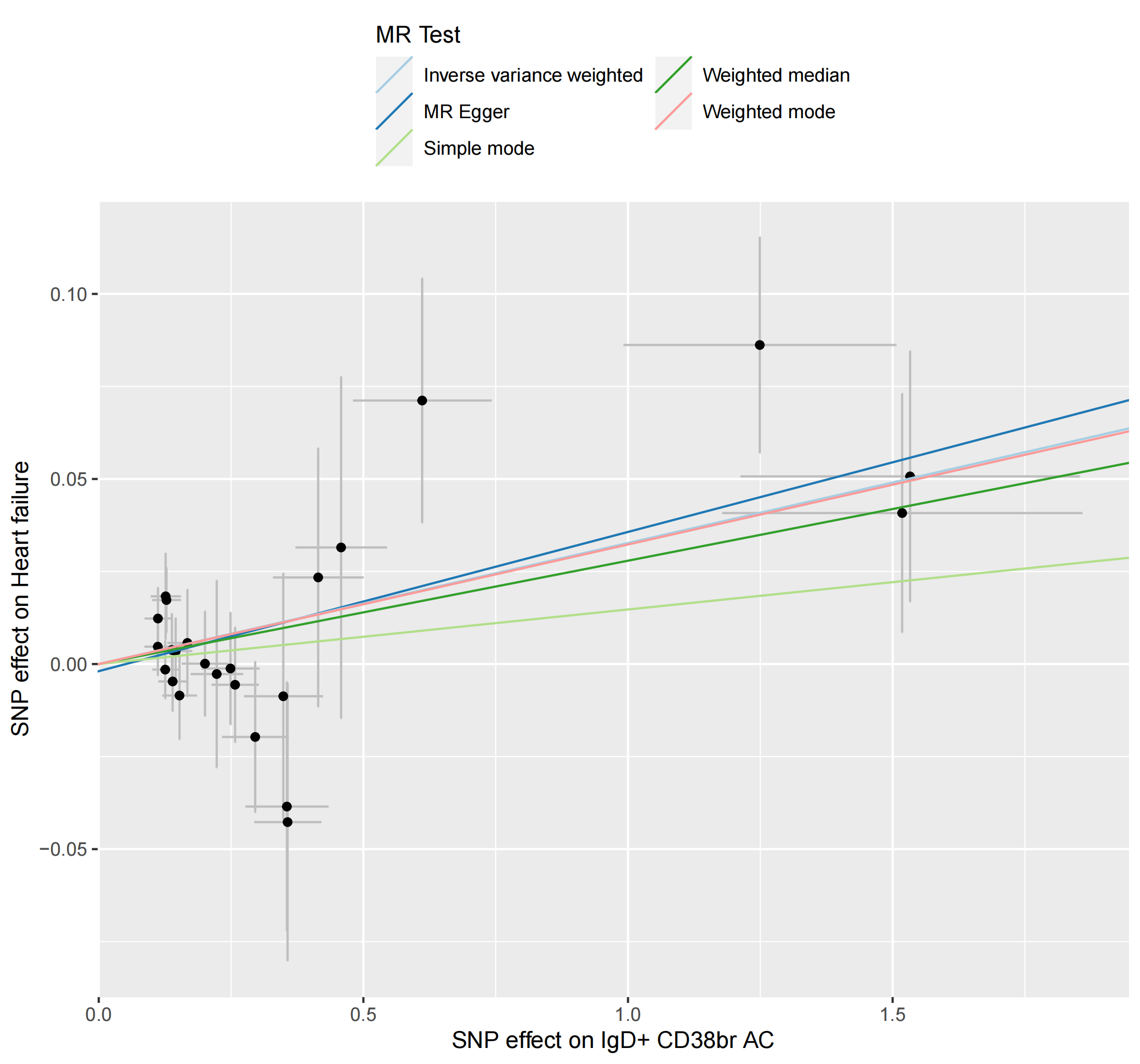 | 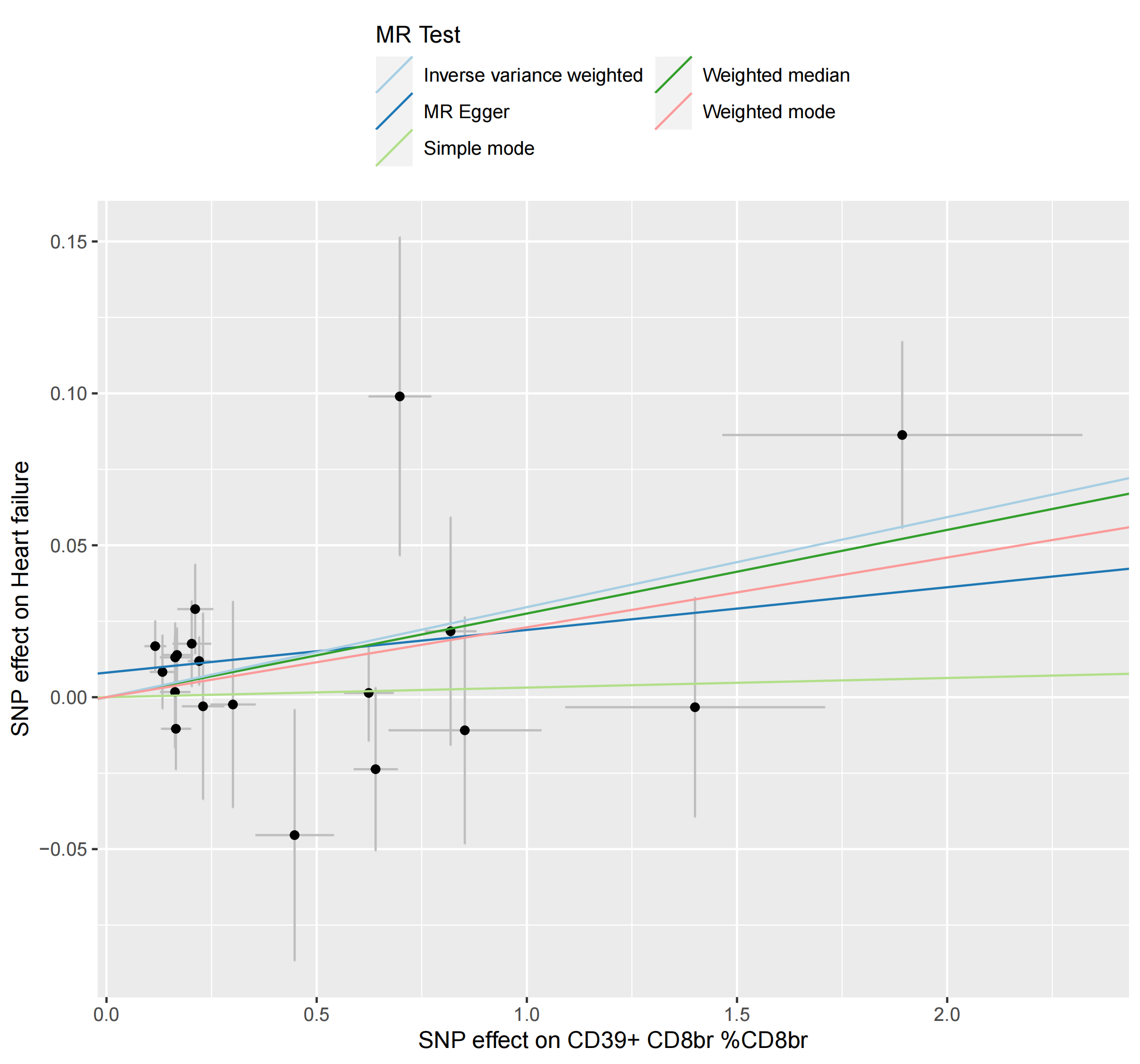 |
| --- | --- |
| 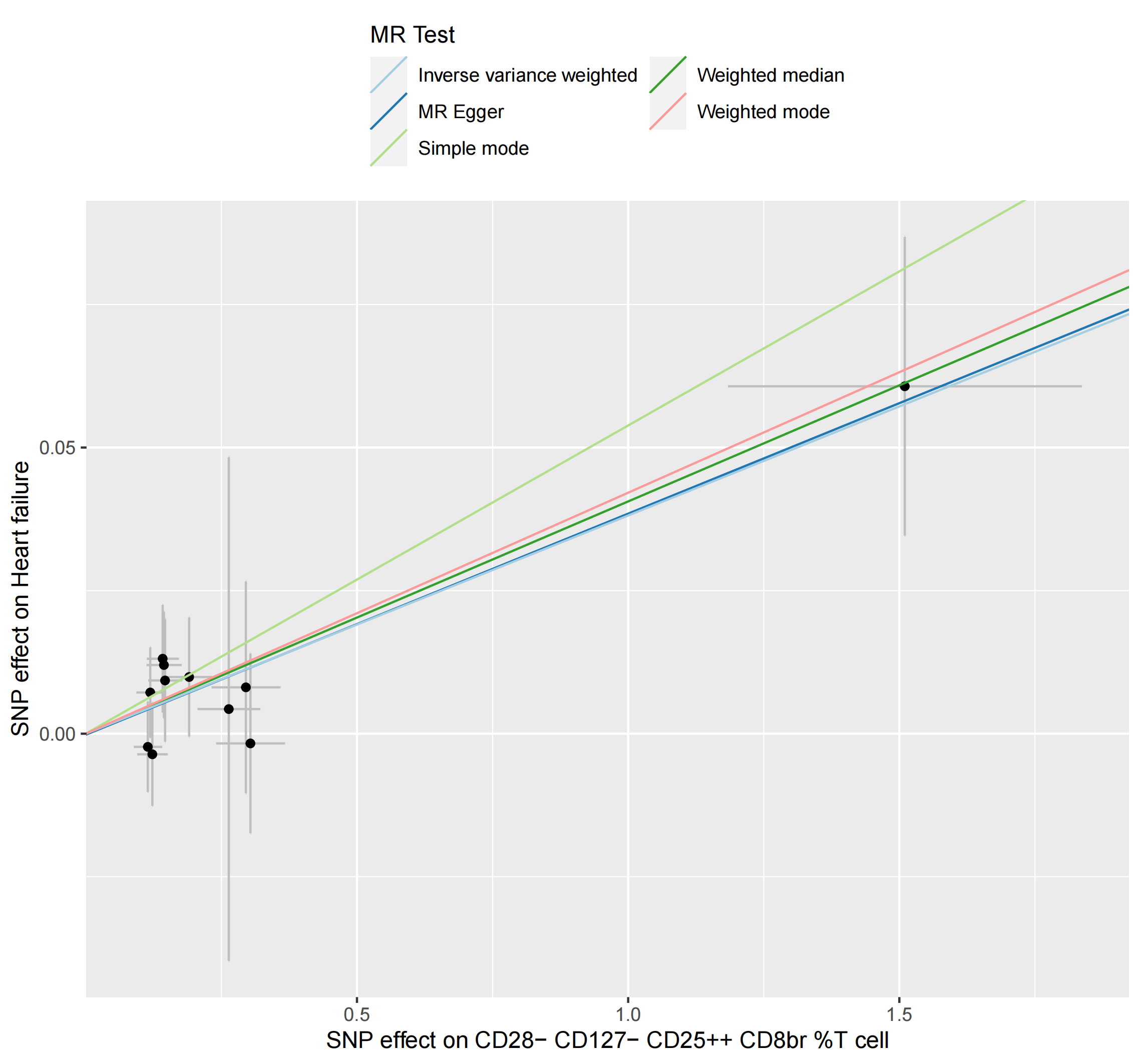 | 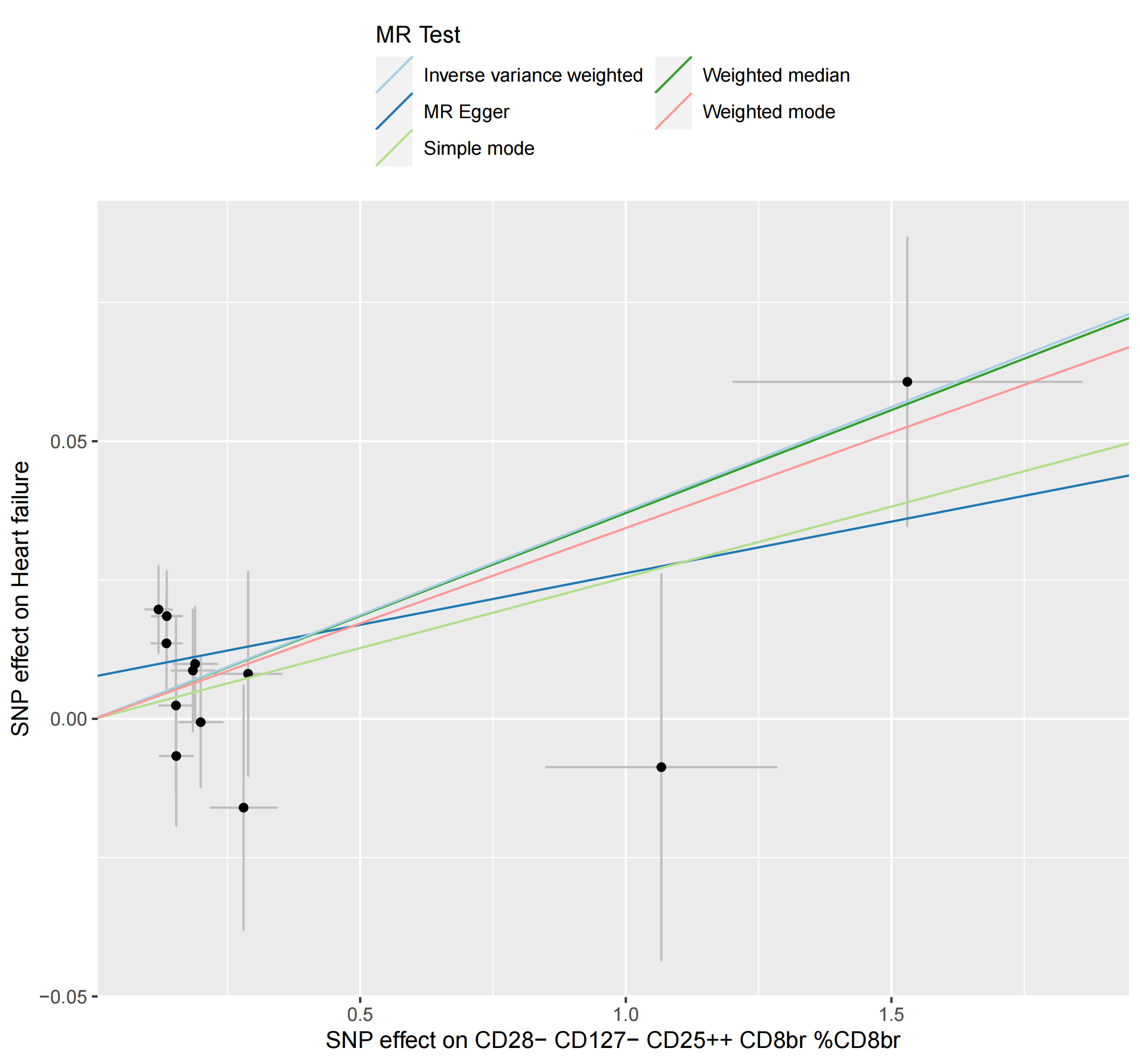 |
| 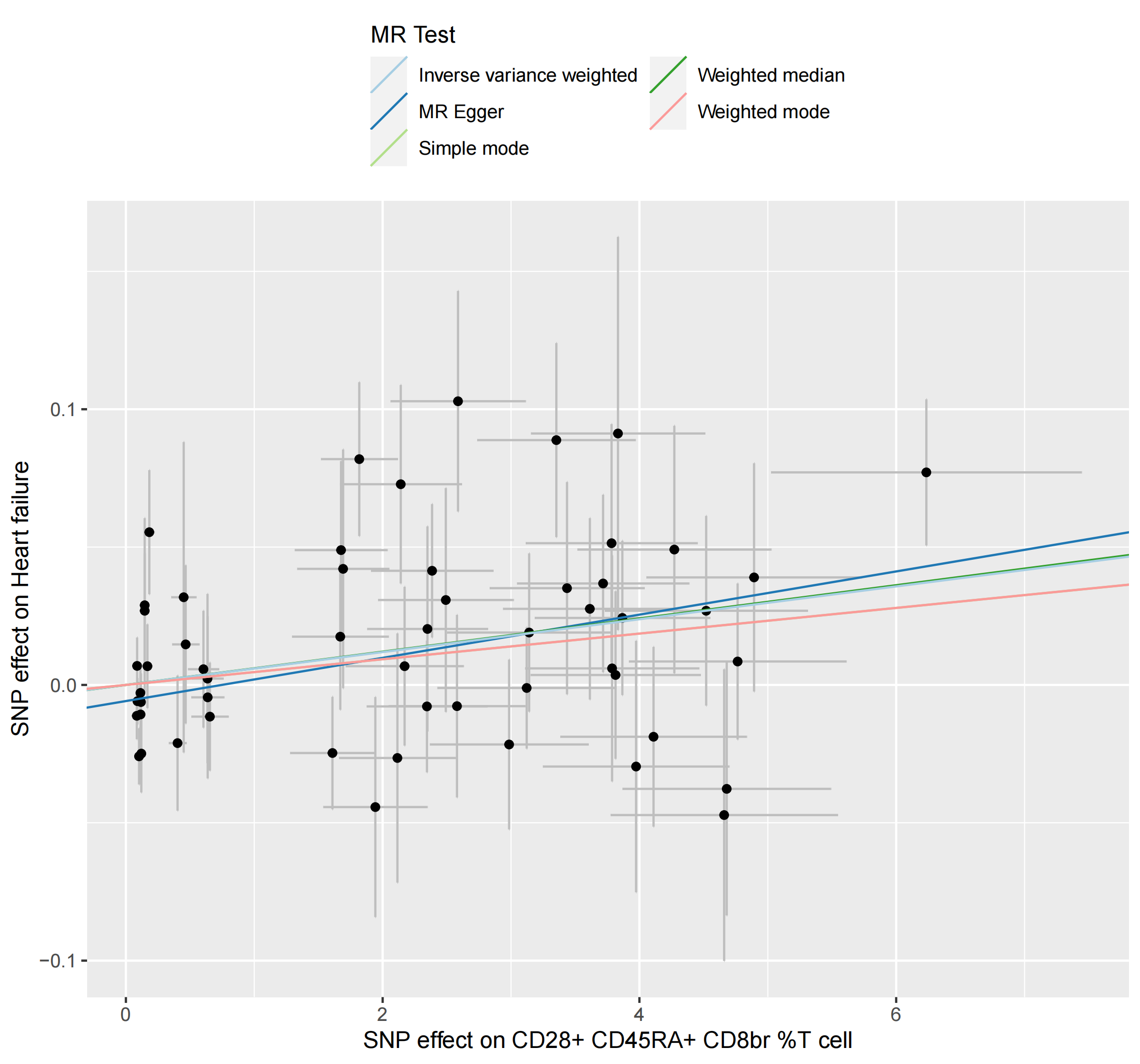 | 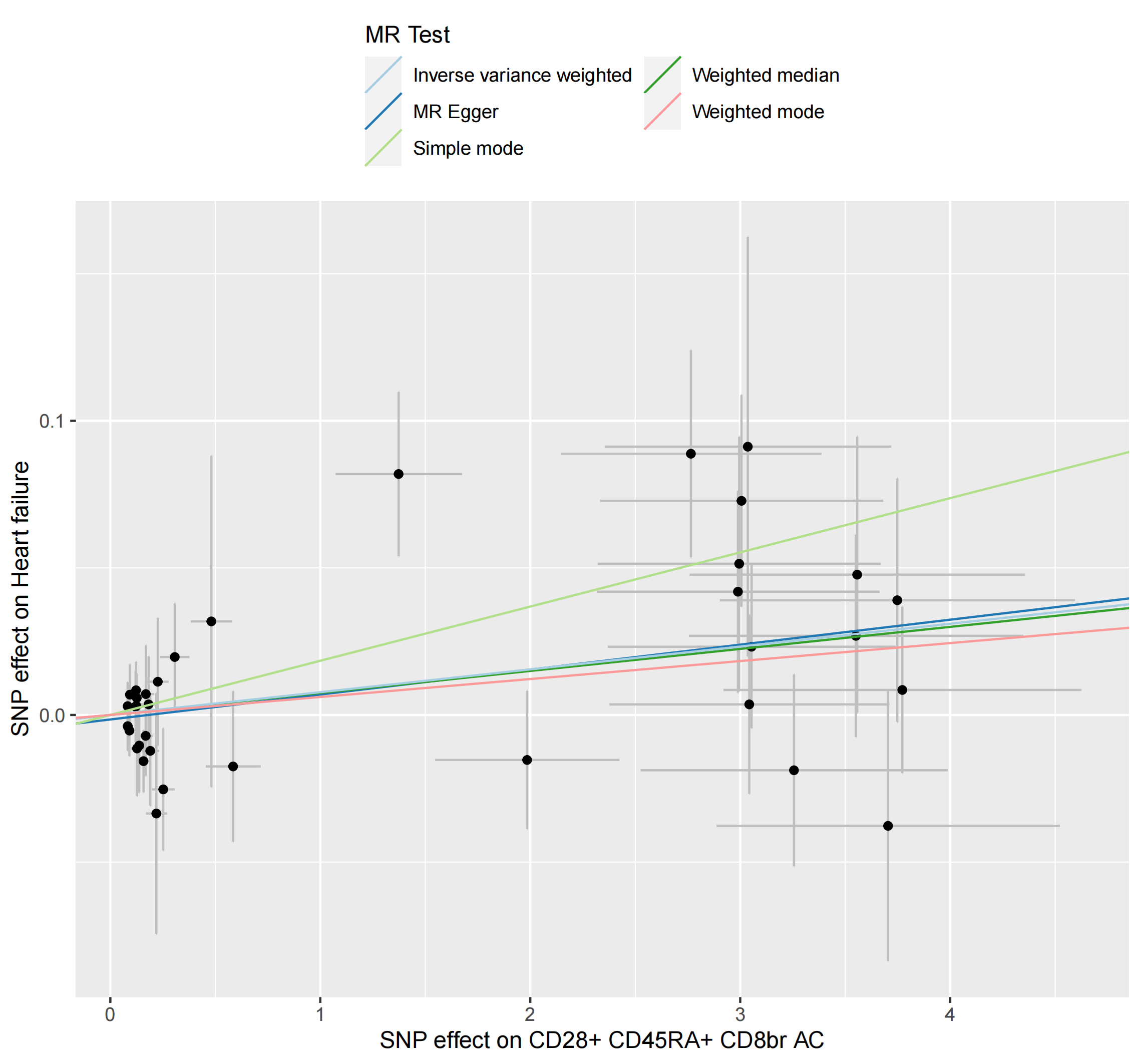 |
| 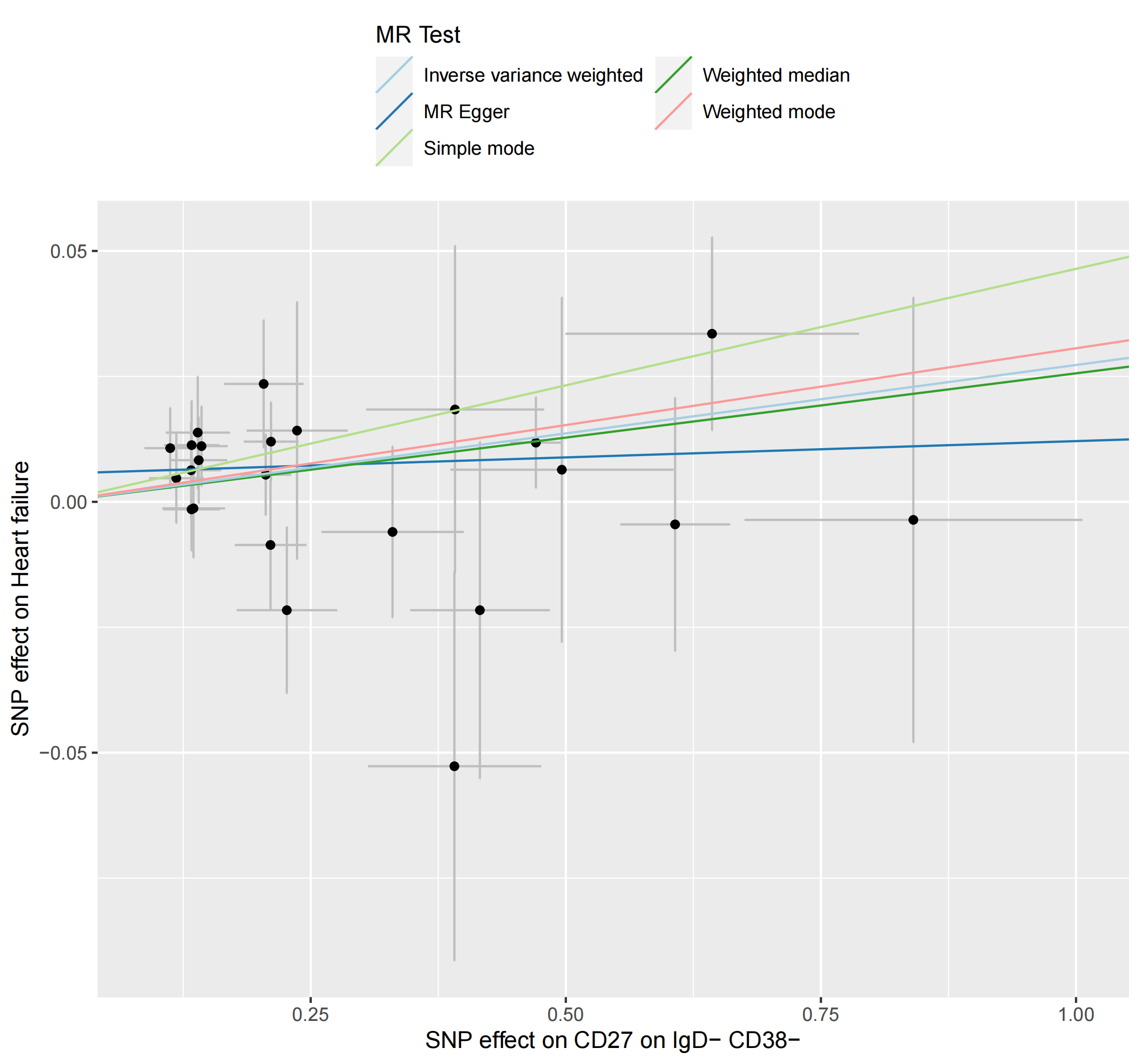 |  |

**Supplementary Figure S2.** The funnel plot in MR studies of Immune cells to Heart failure (*P*<0.01).

| 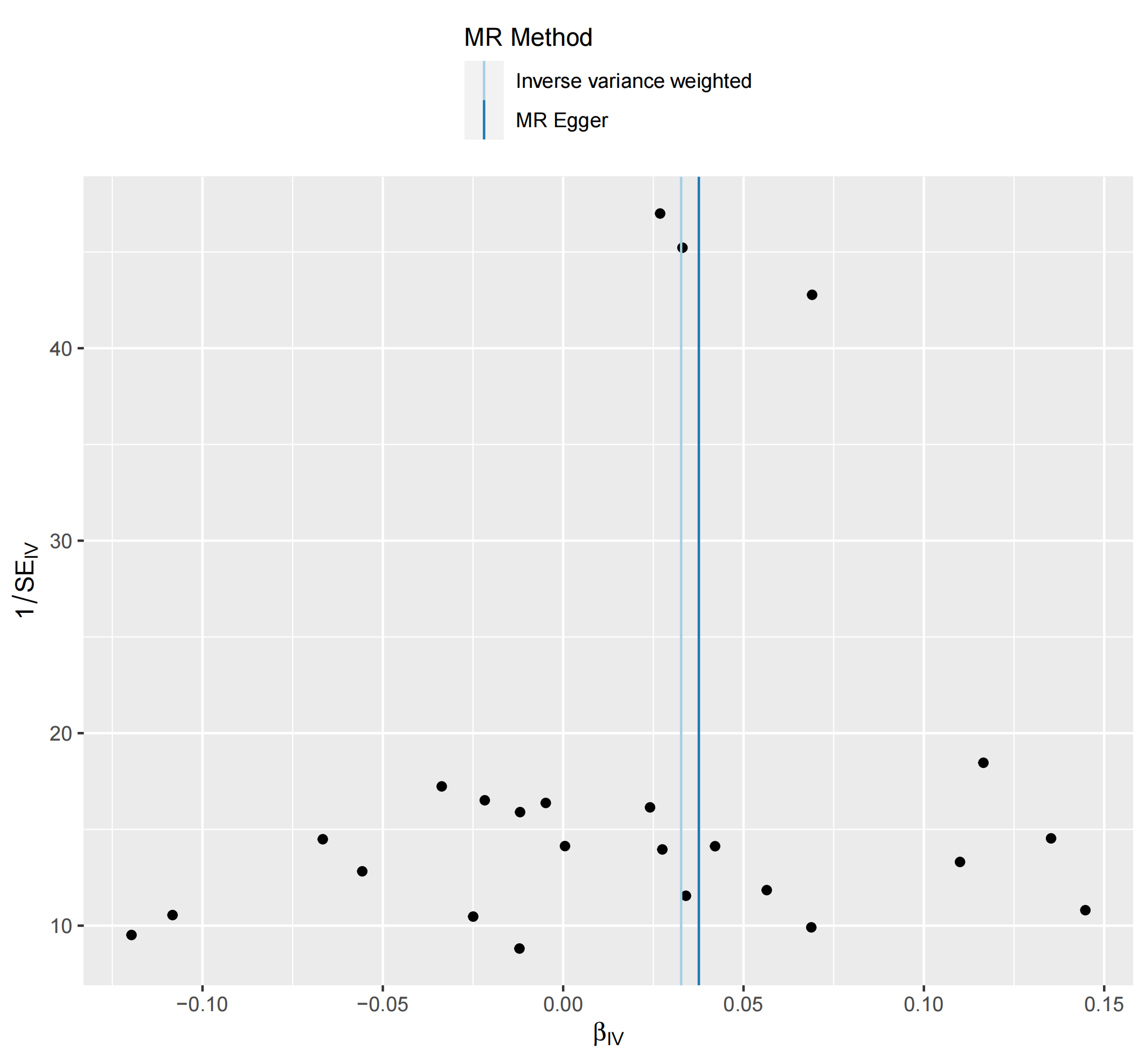 | 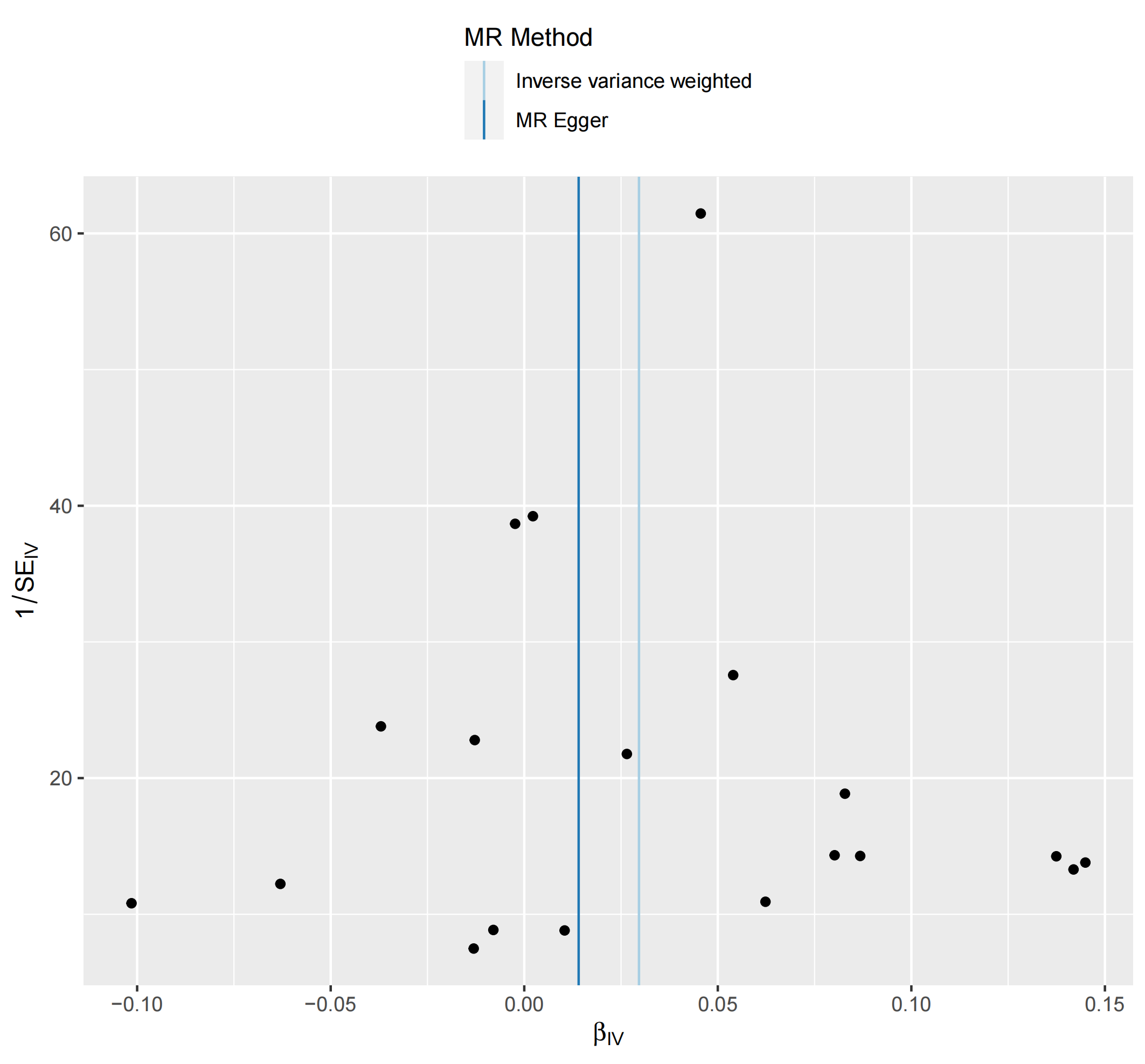 |
| --- | --- |
| 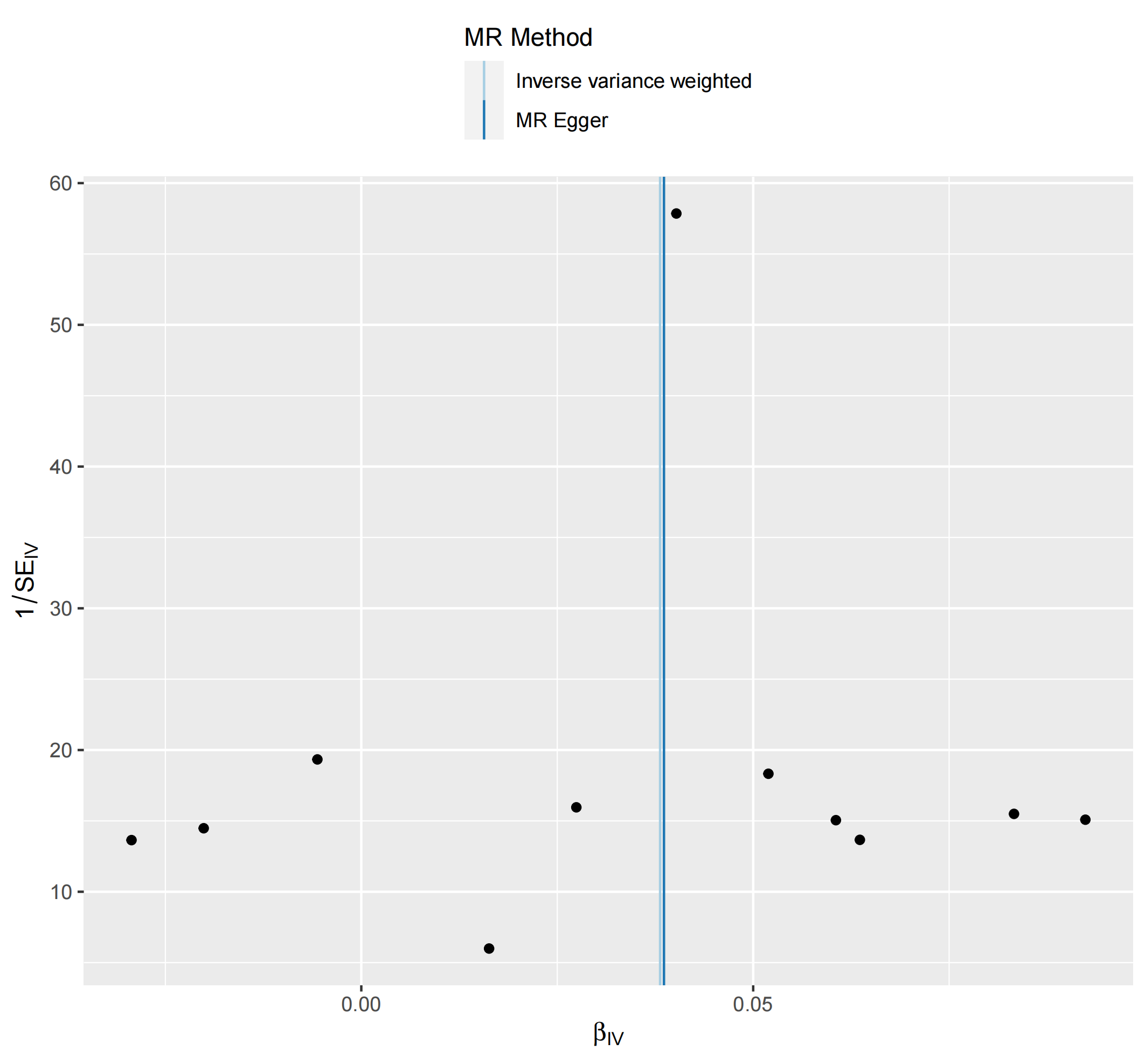 | 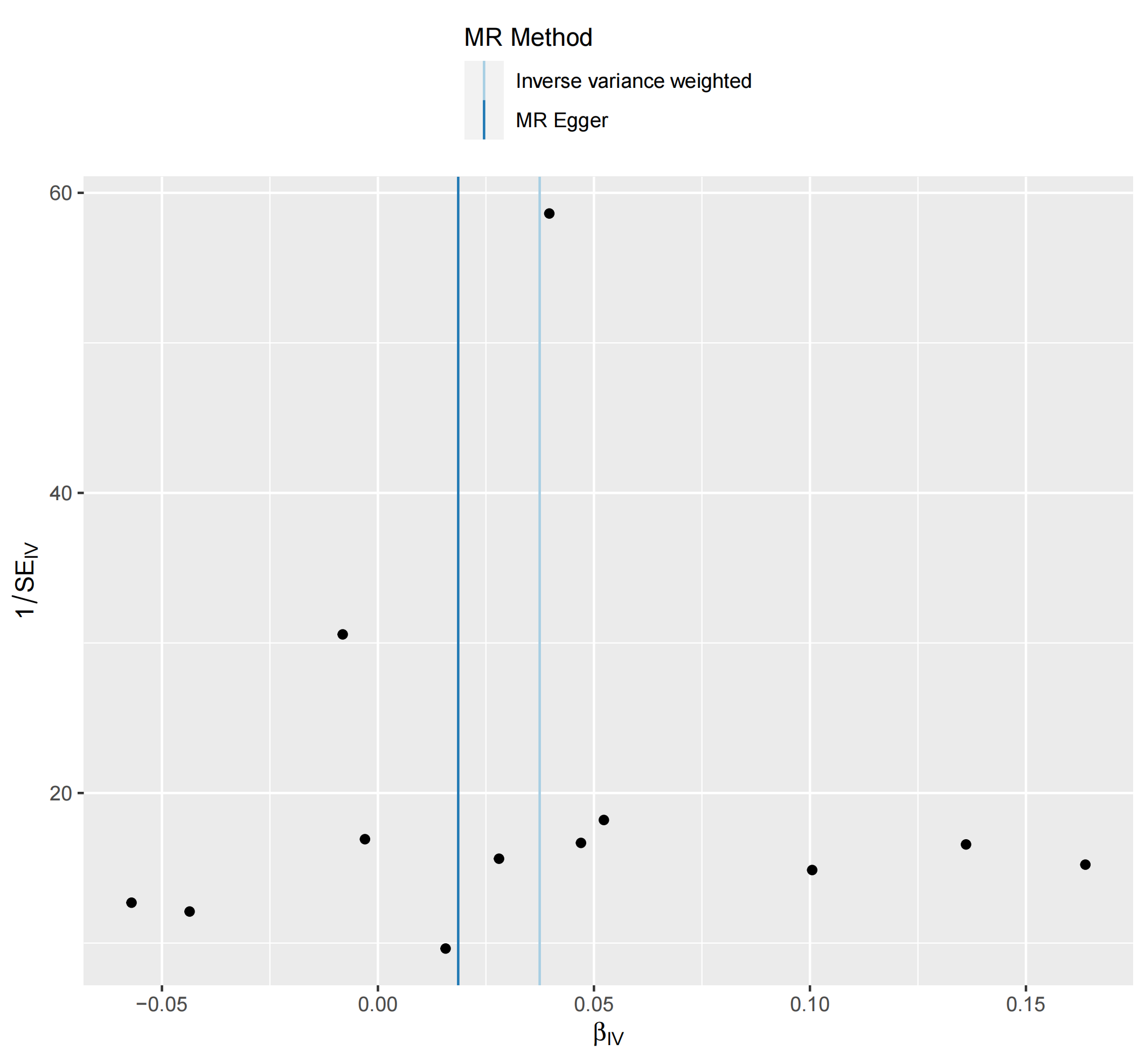 |
| 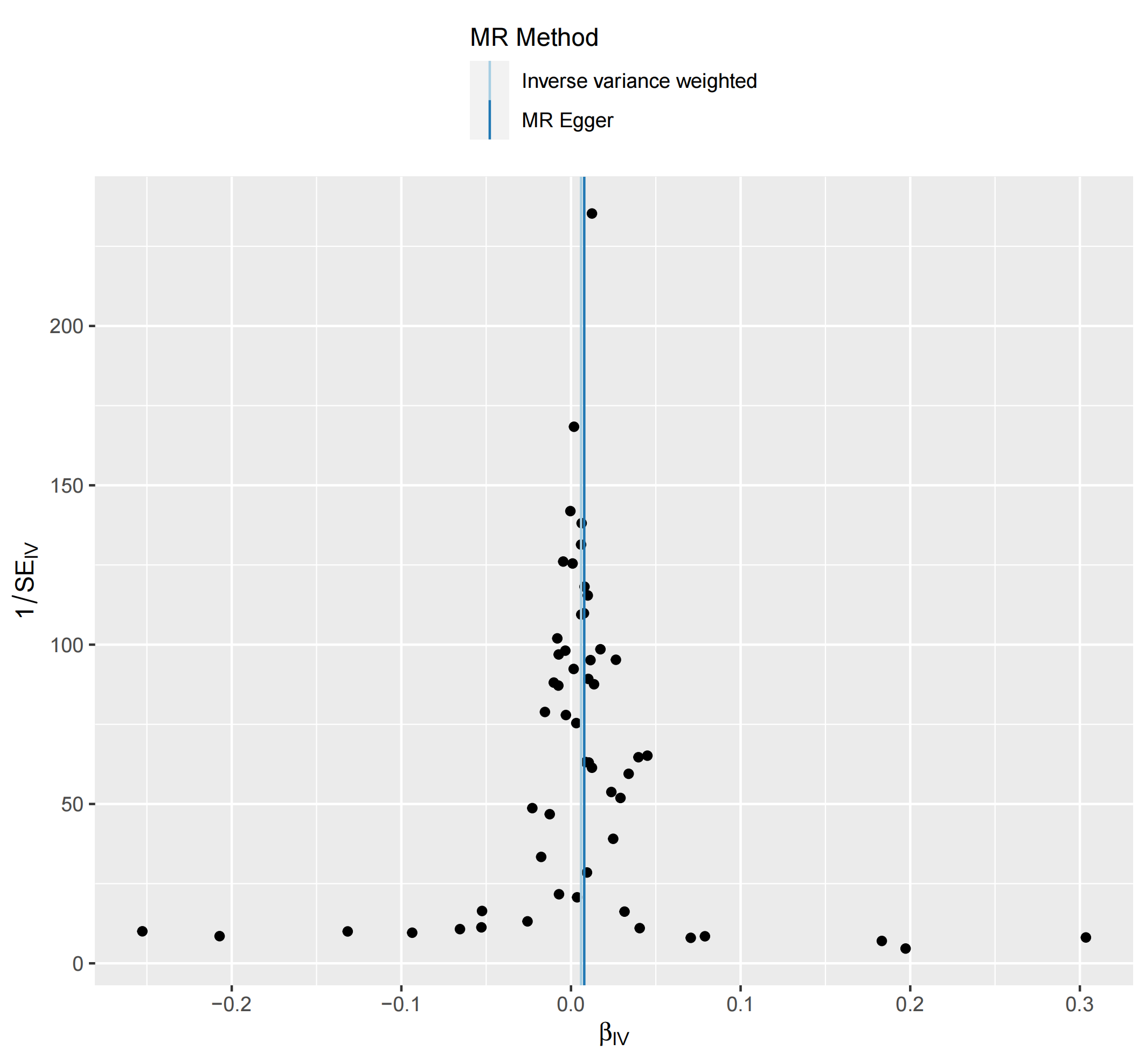 | 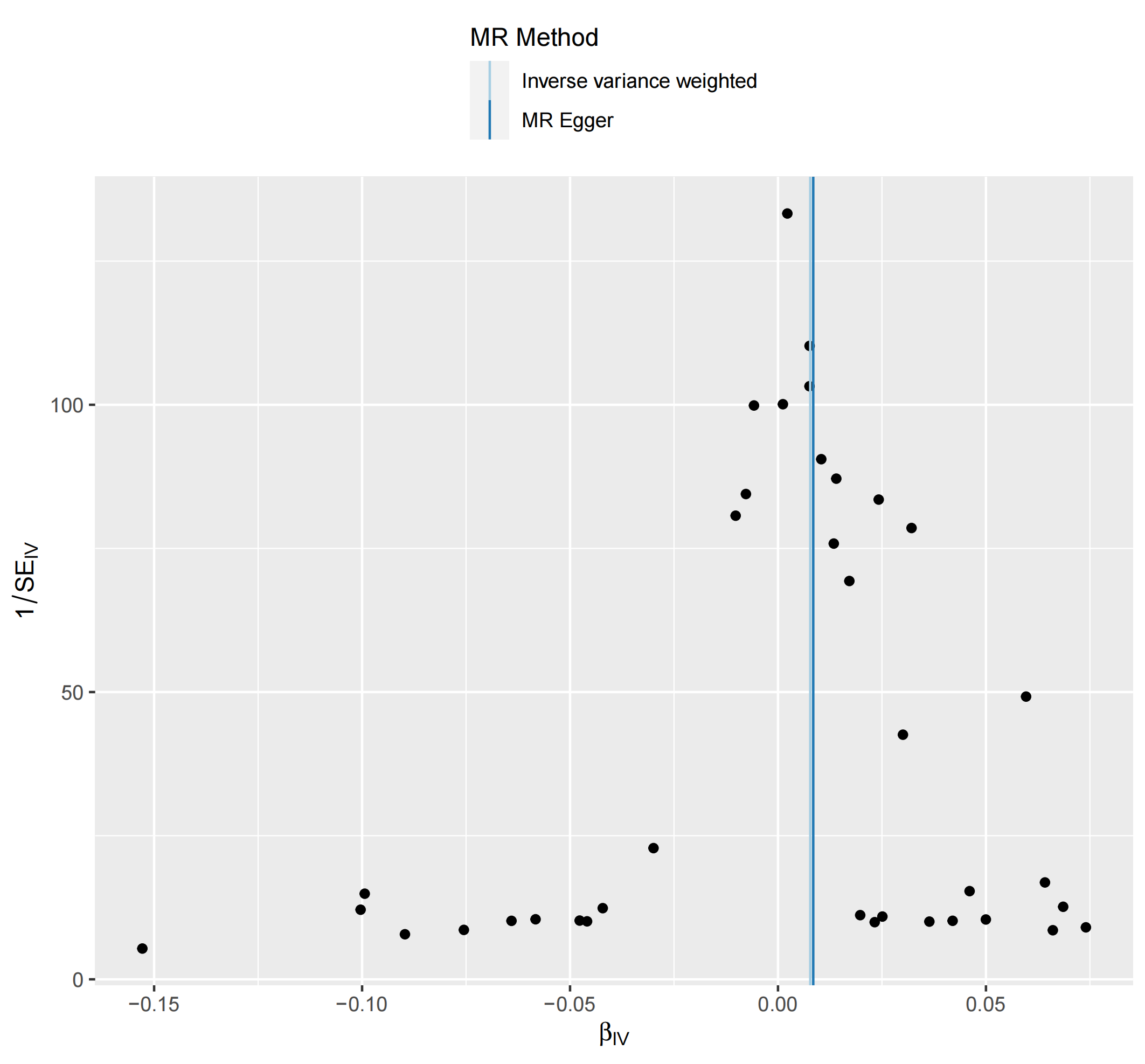 |
| 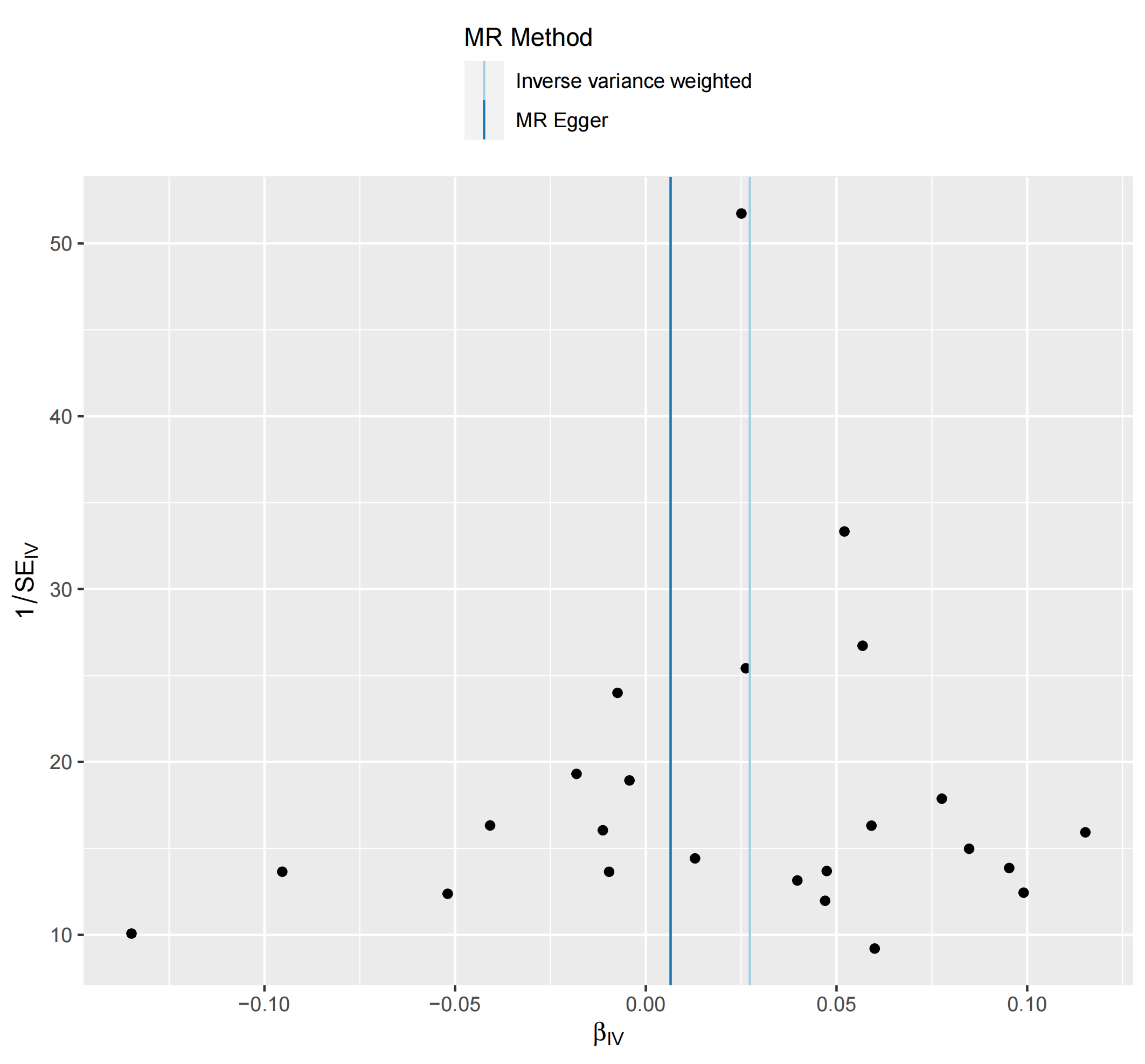 |  |

**Supplementary Figure S3.** The leave-one-out analysis results in MR studies of Immune cells to Heart failure (*P*<0.01).

| 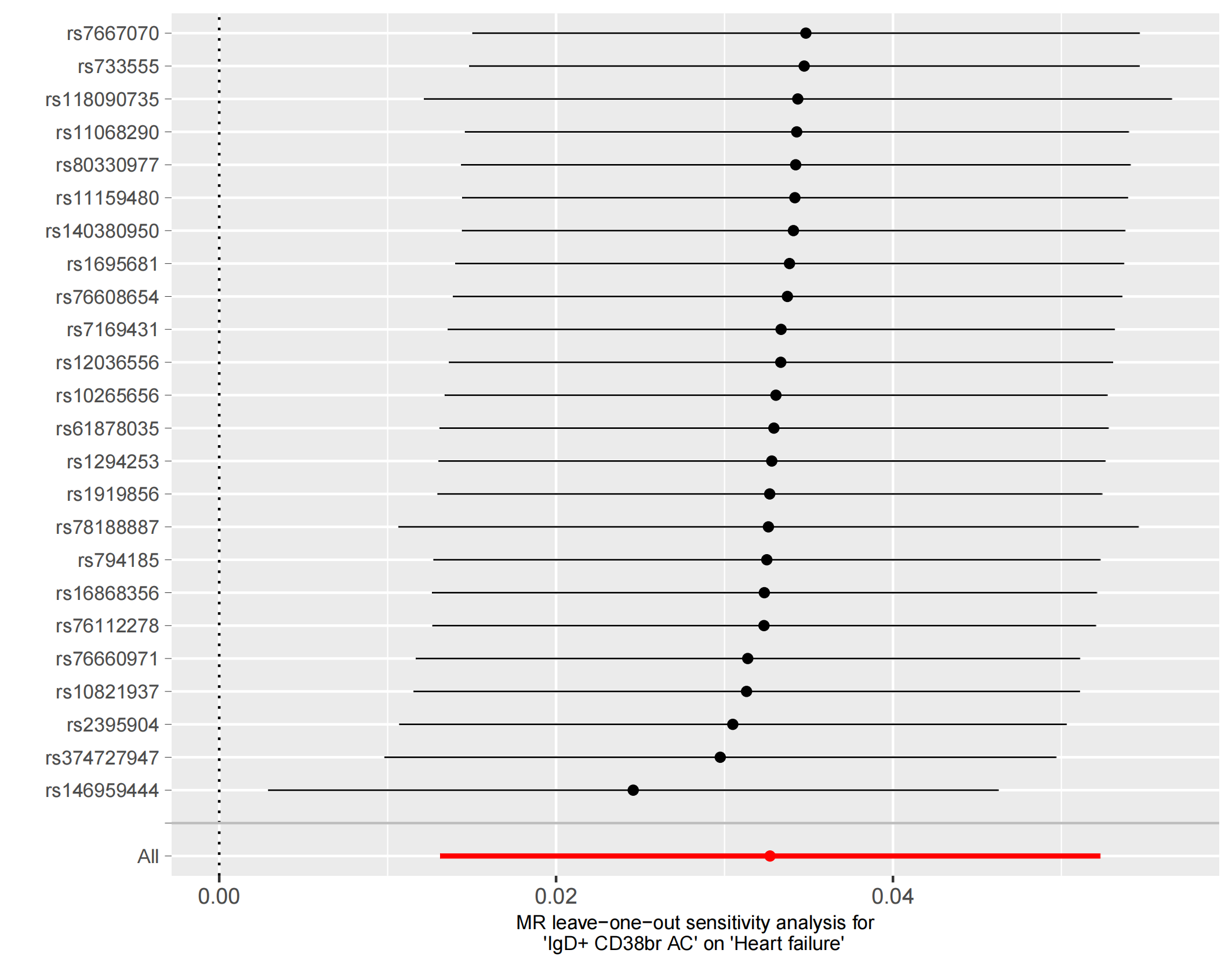 | 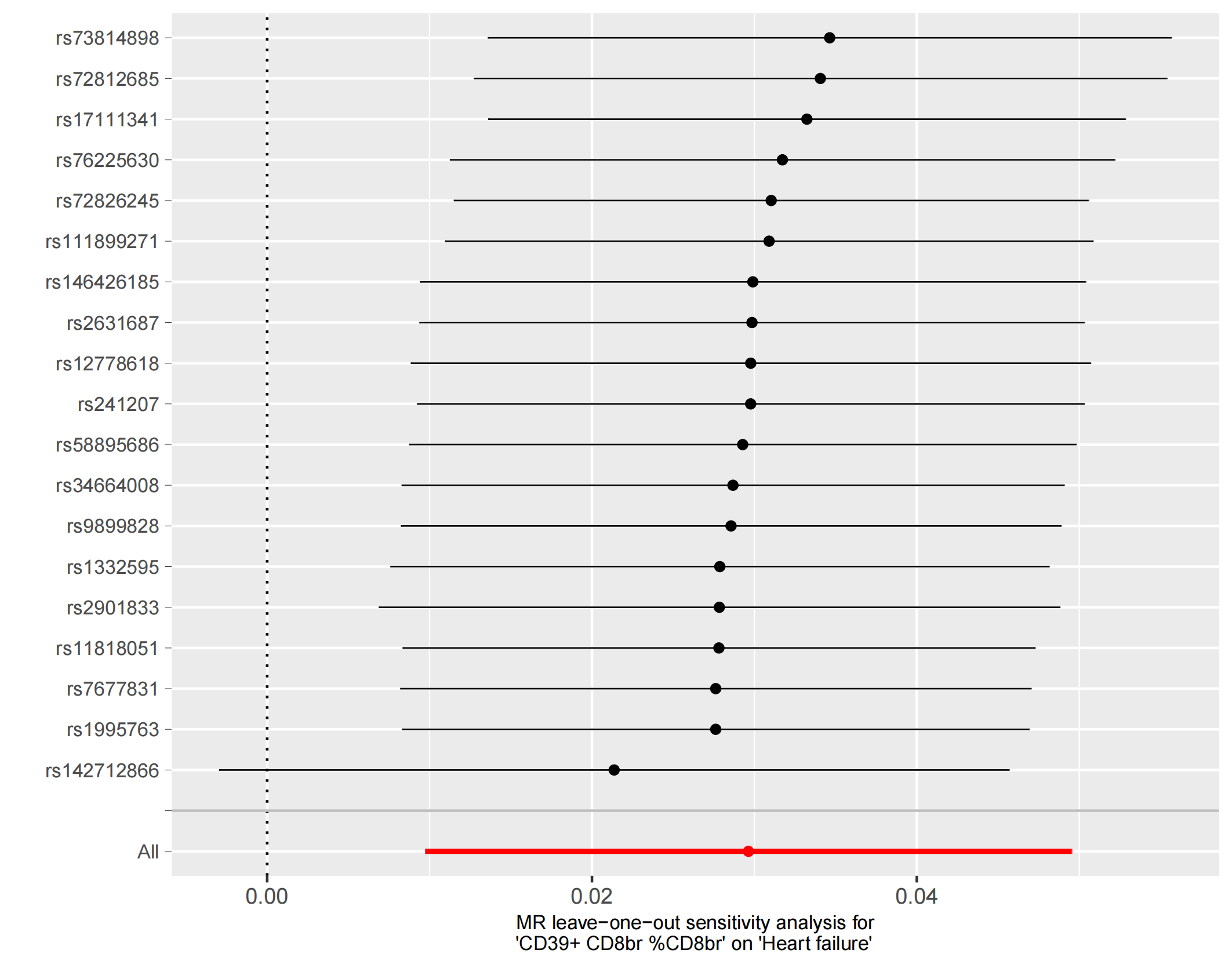 |
| --- | --- |
| 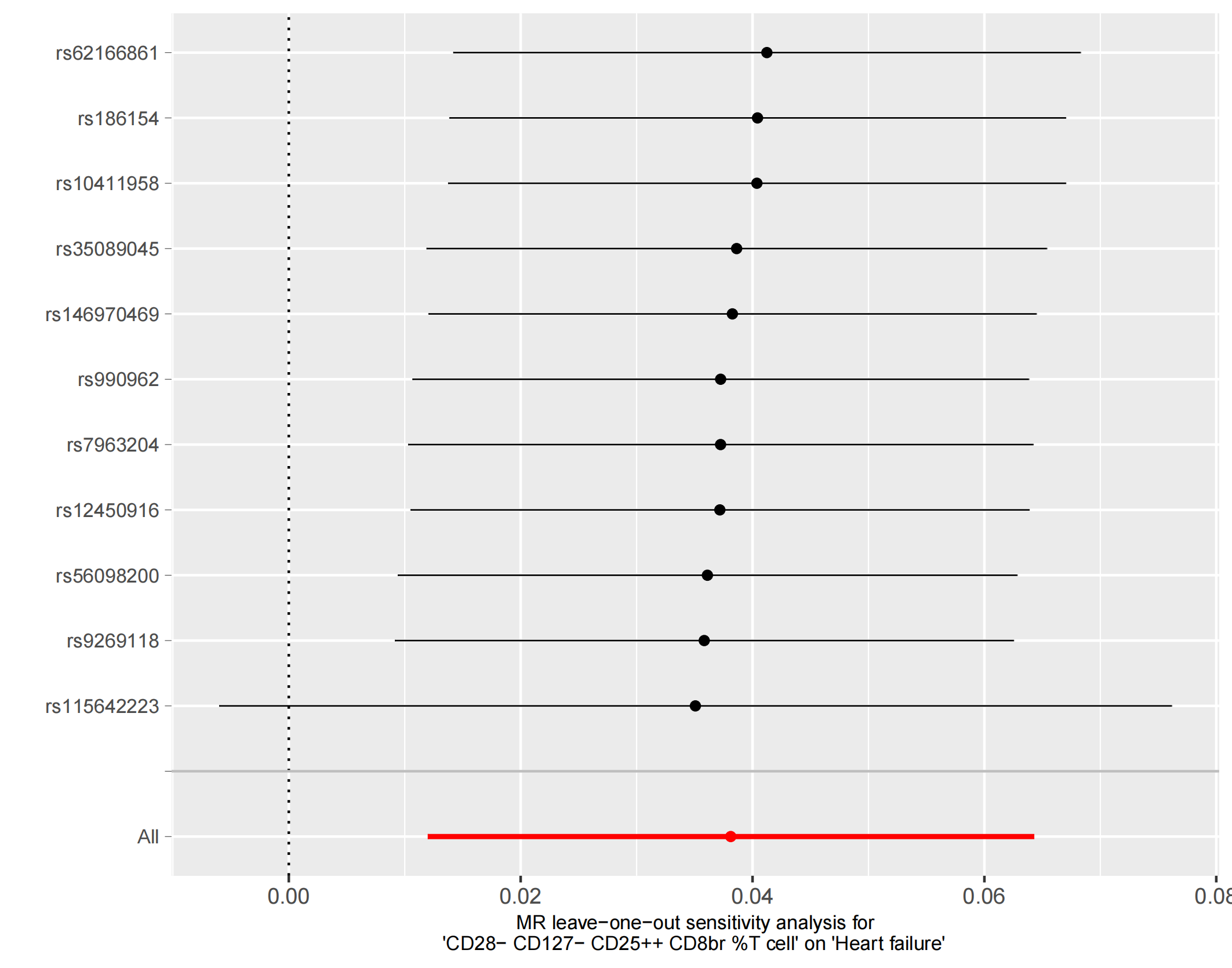 | 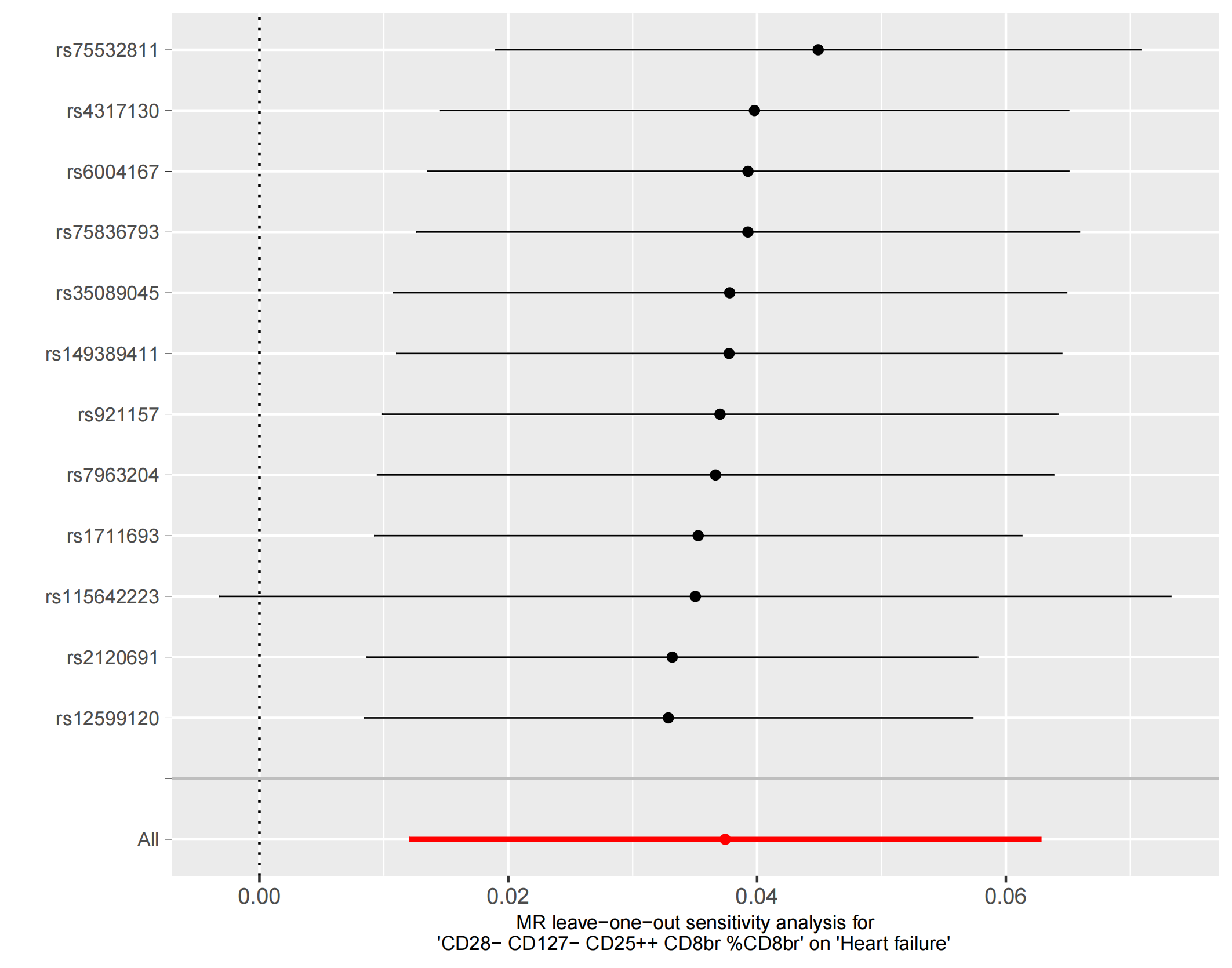 |
| 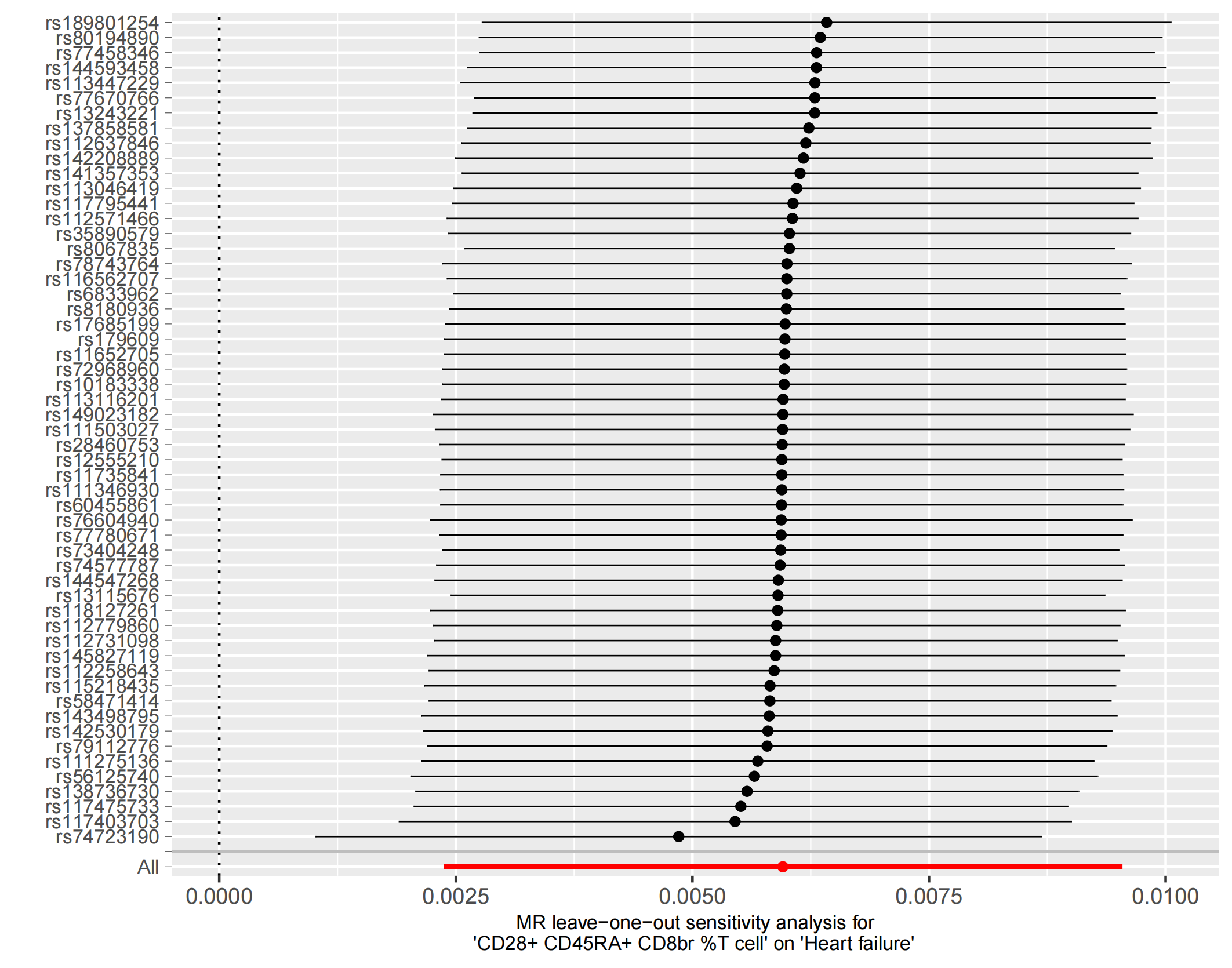 | 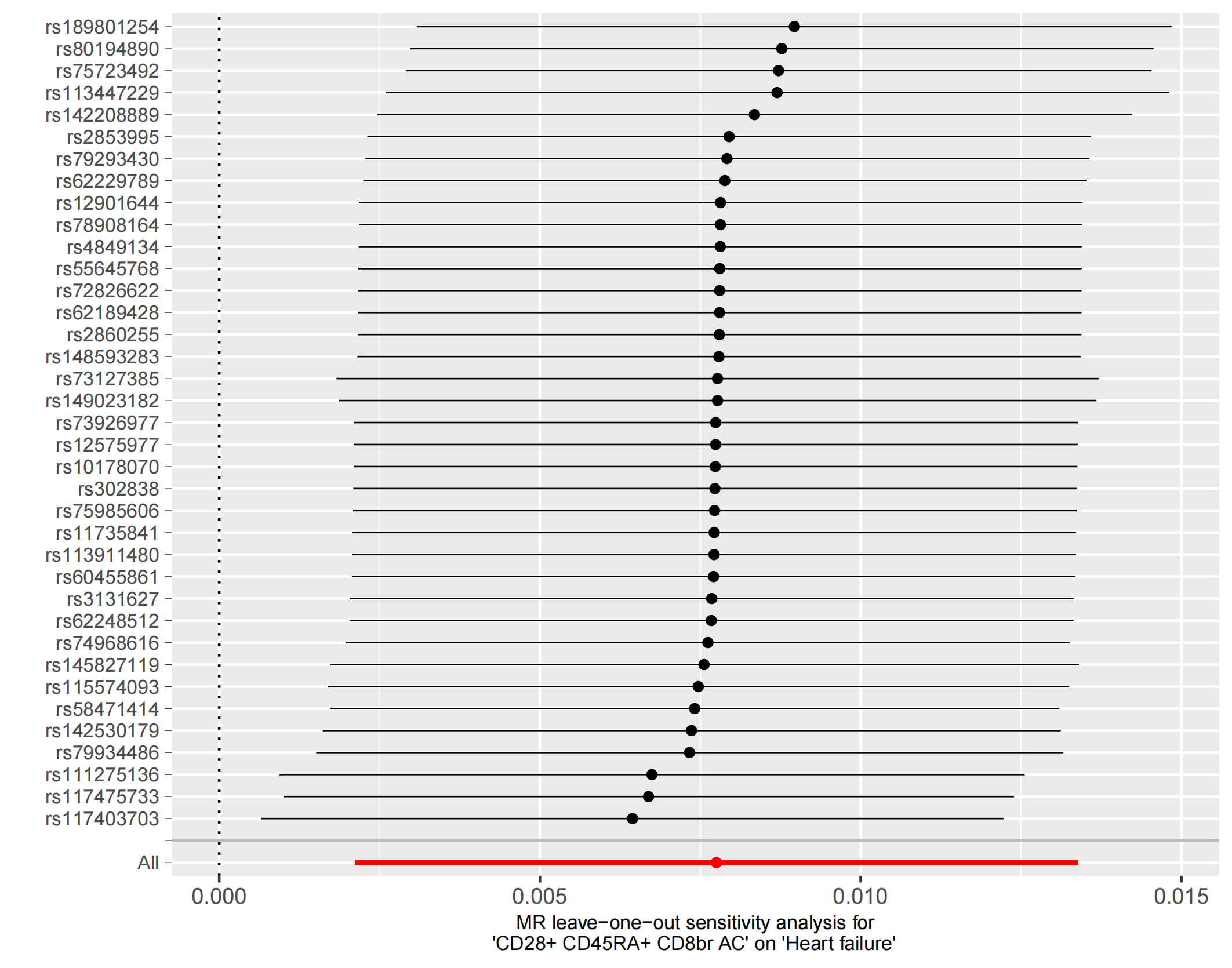 |
| 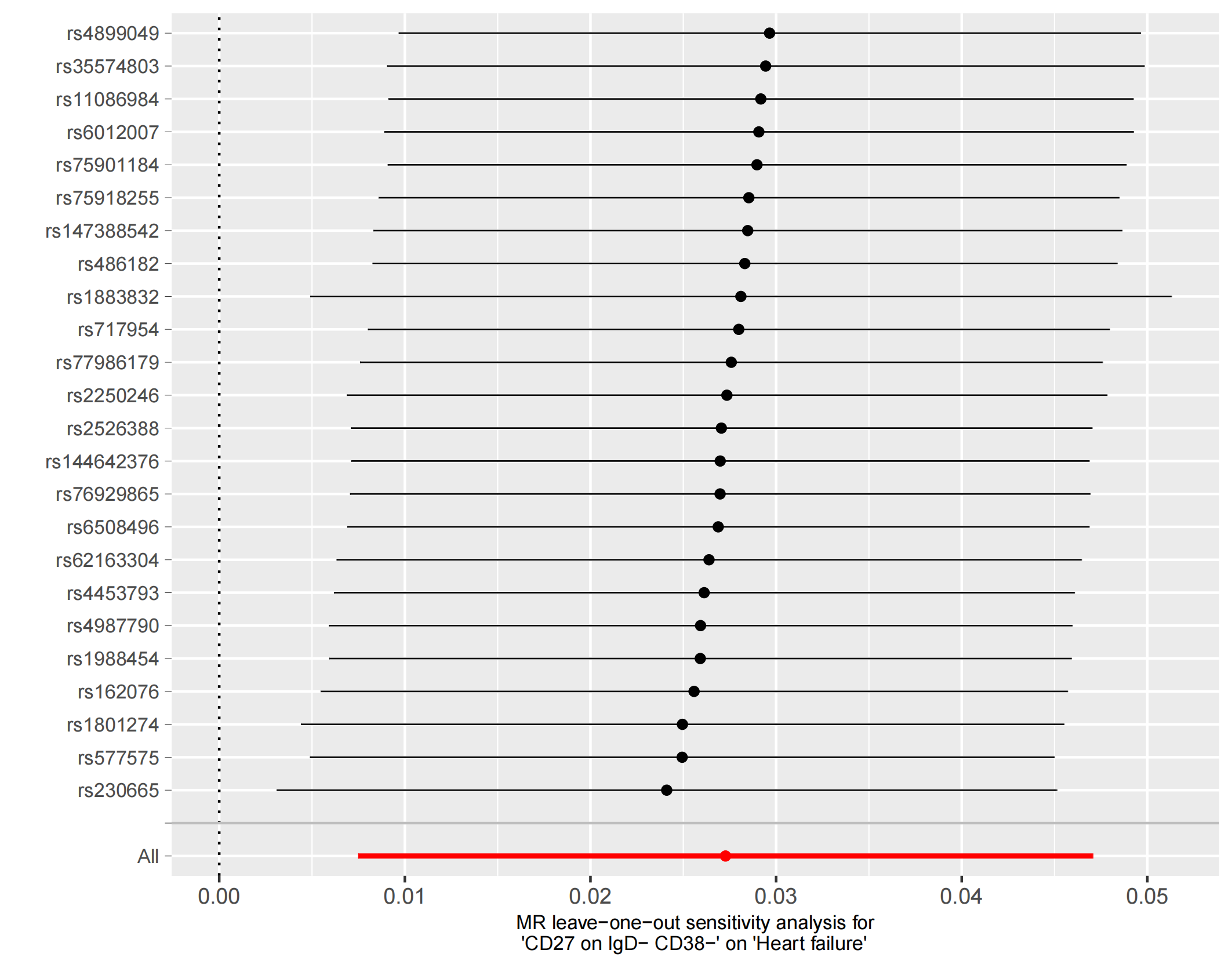 |  |

**Supplementary Figure S4.** The scatter plot in MR studies of Plasma Metabolites to Heart failure (*P*<0.01).

| 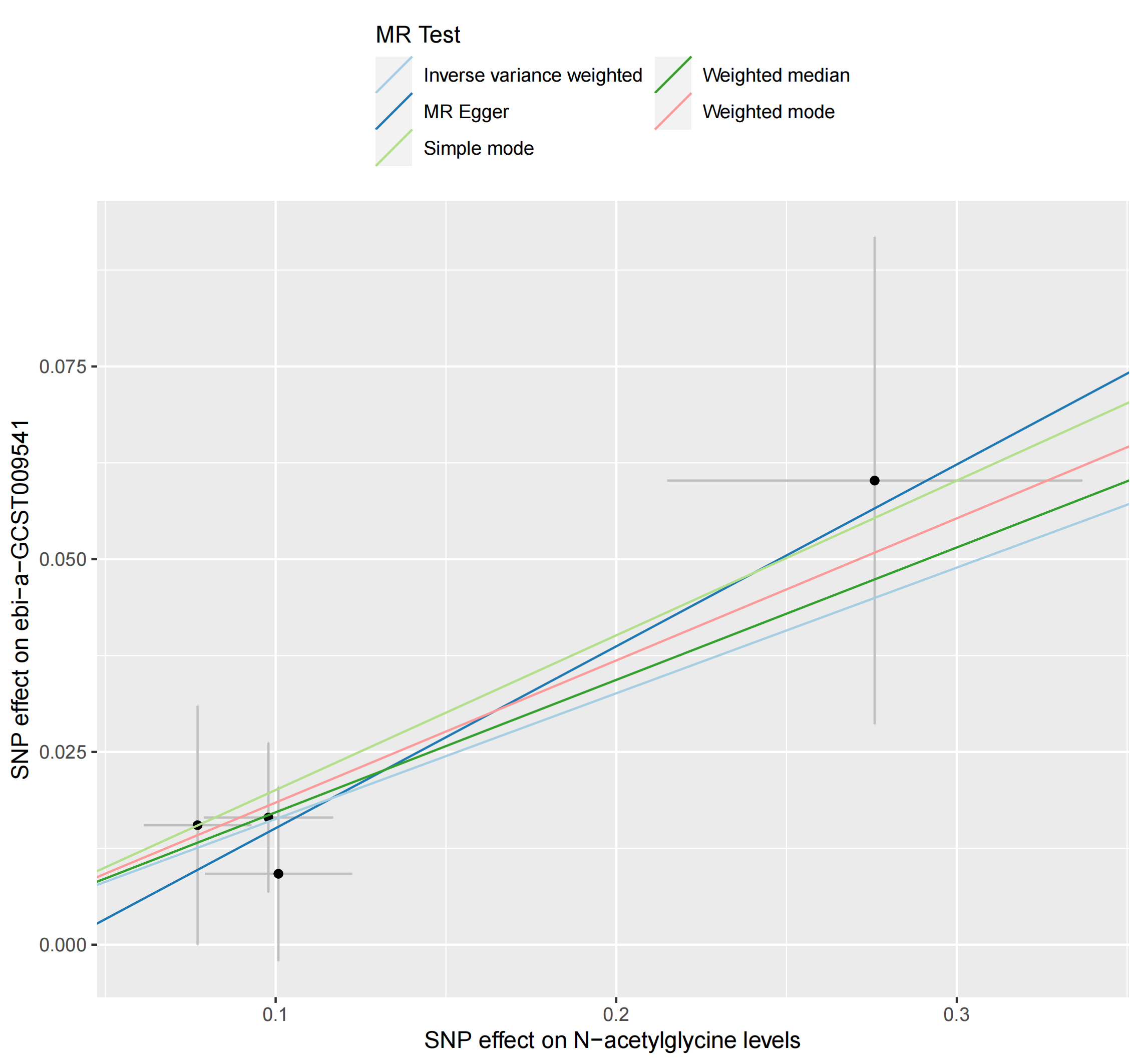 | 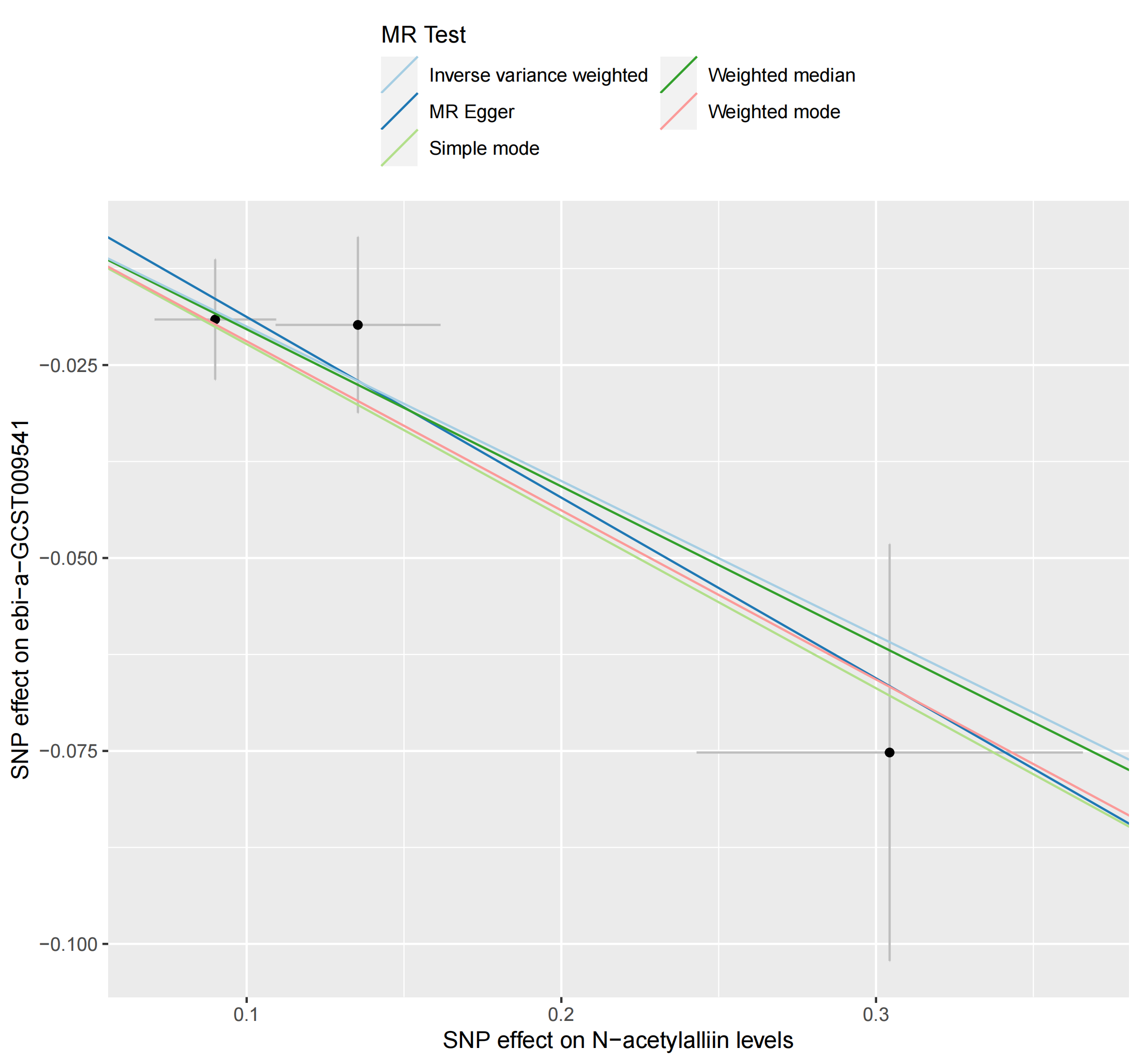 |
| --- | --- |
| 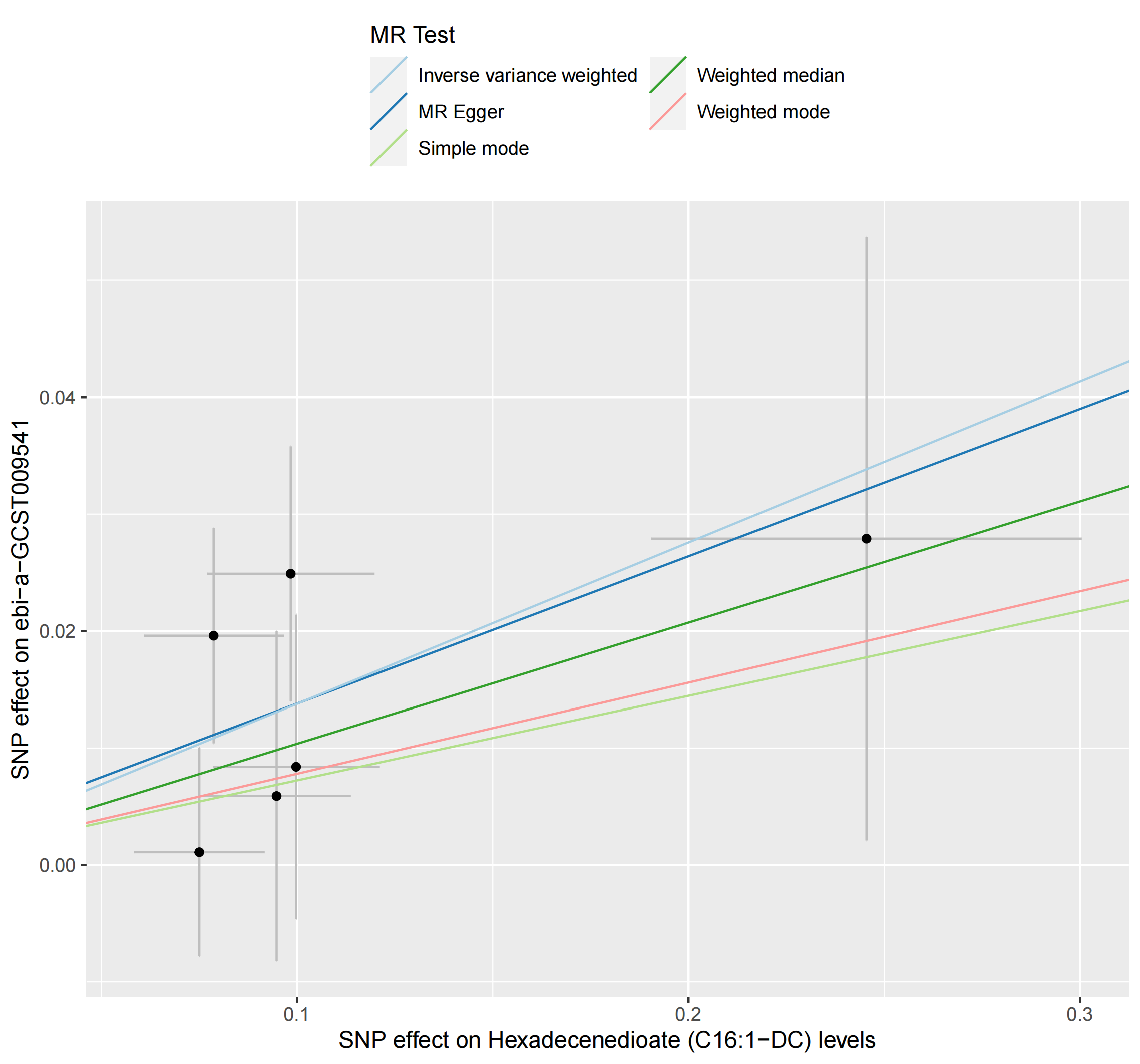 |  |

**Supplementary Figure S5.** The funnel plot in MR studies of Plasma Metabolites to Heart failure (*P*<0.01).

| 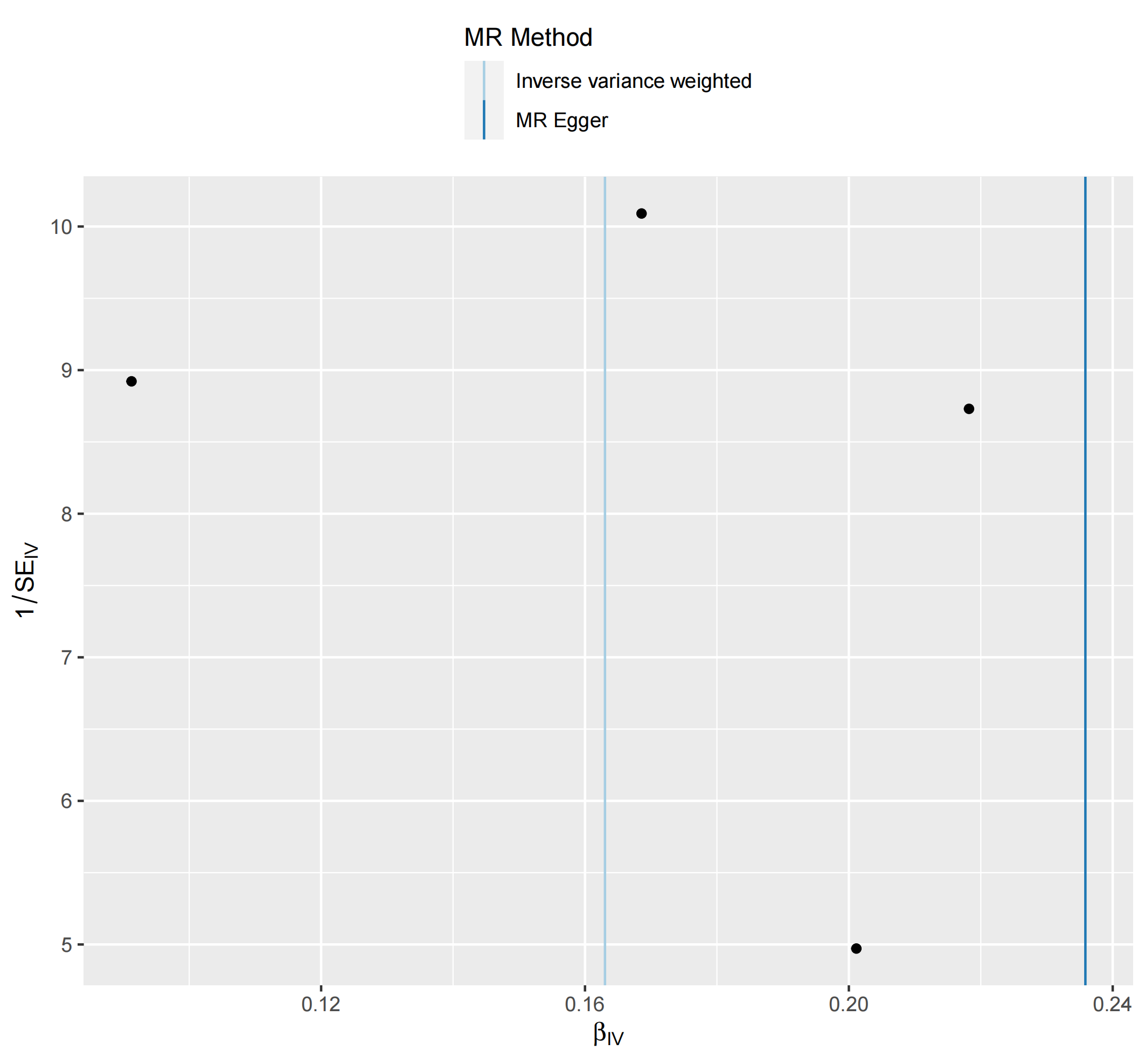 | 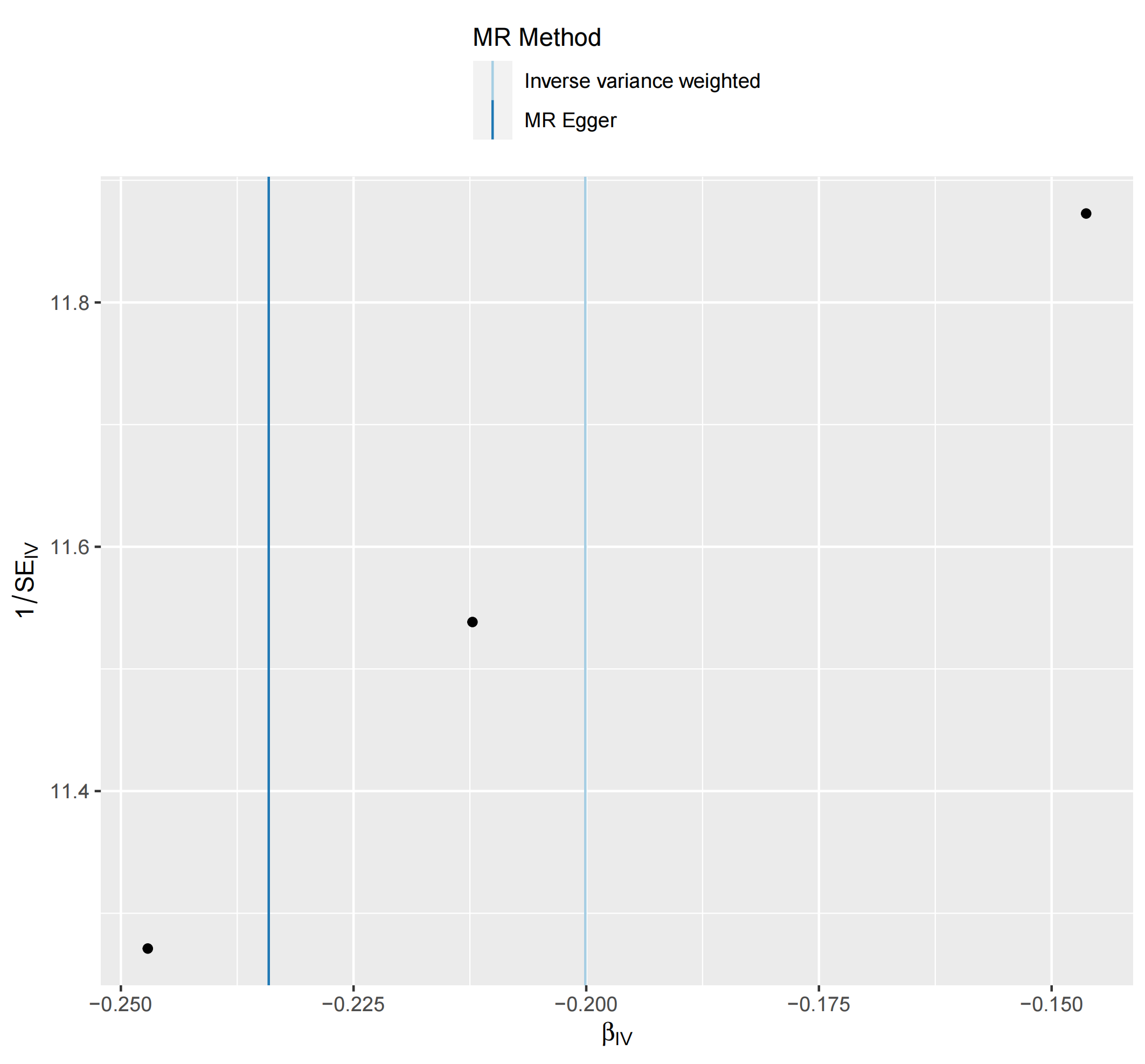 |
| --- | --- |
| 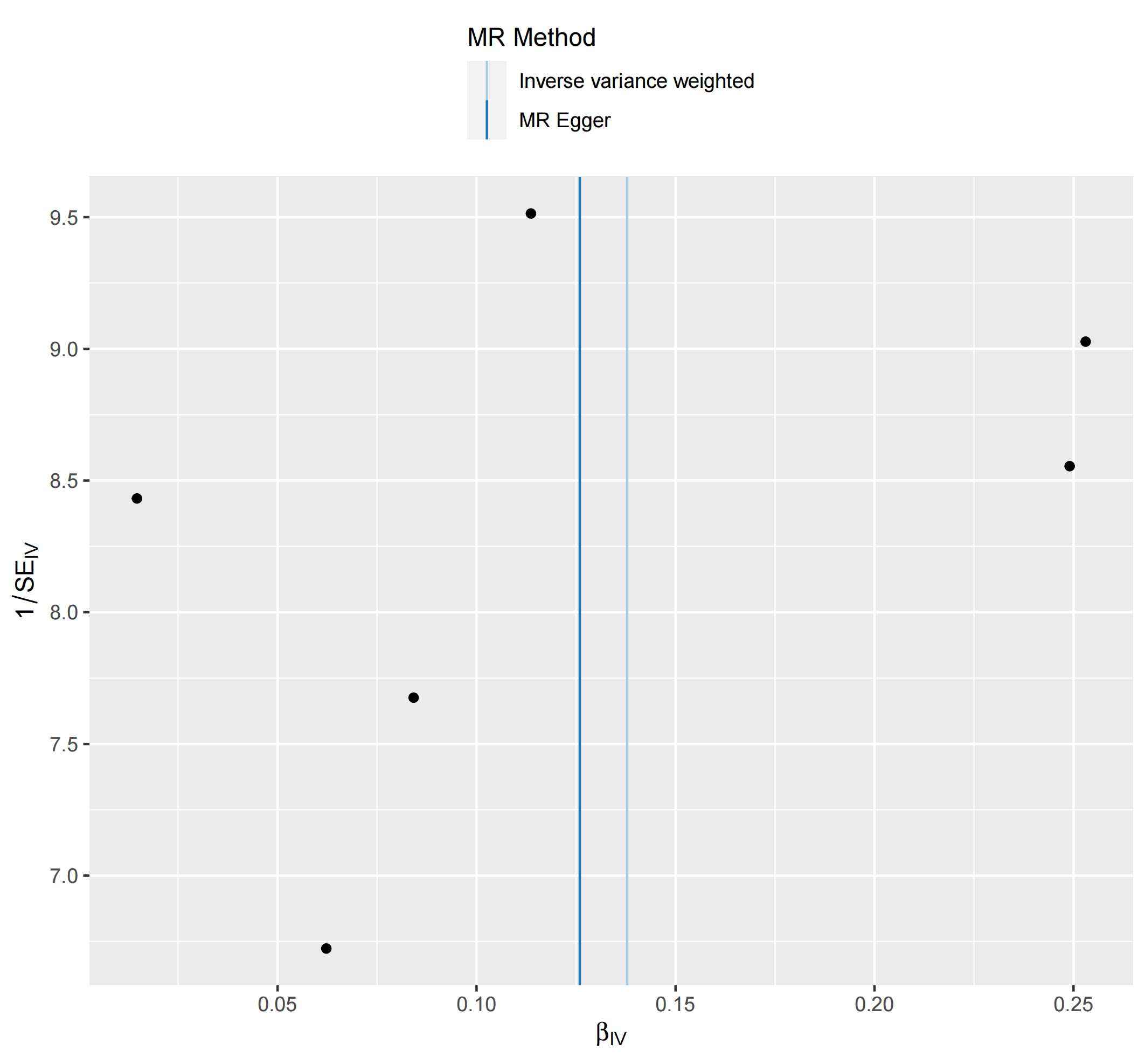 |  |

**Supplementary Figure S6.** The leave-one-out analysis results in MR studies of Plasma Metabolites to Heart failure (*P*<0.01).

| 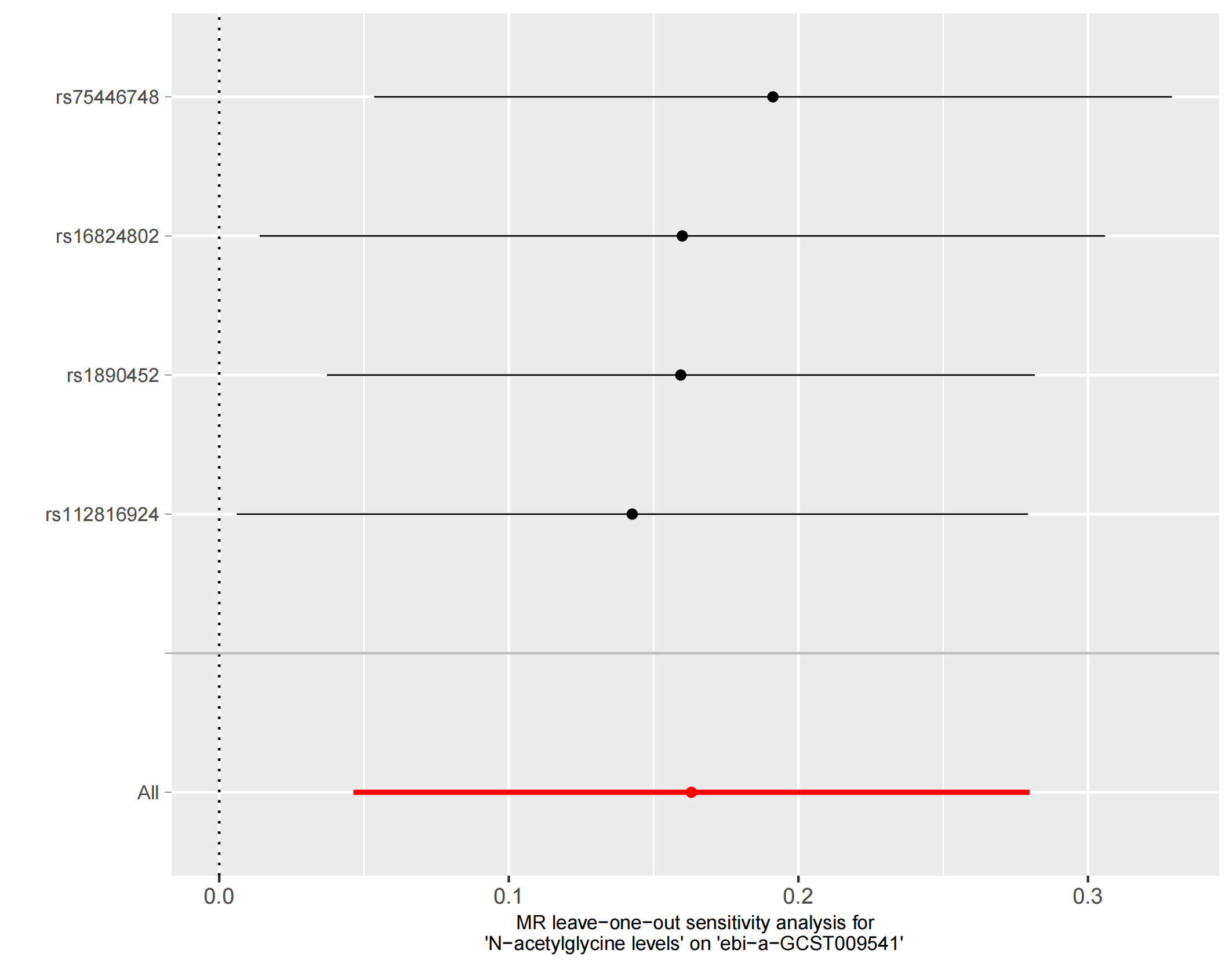 | 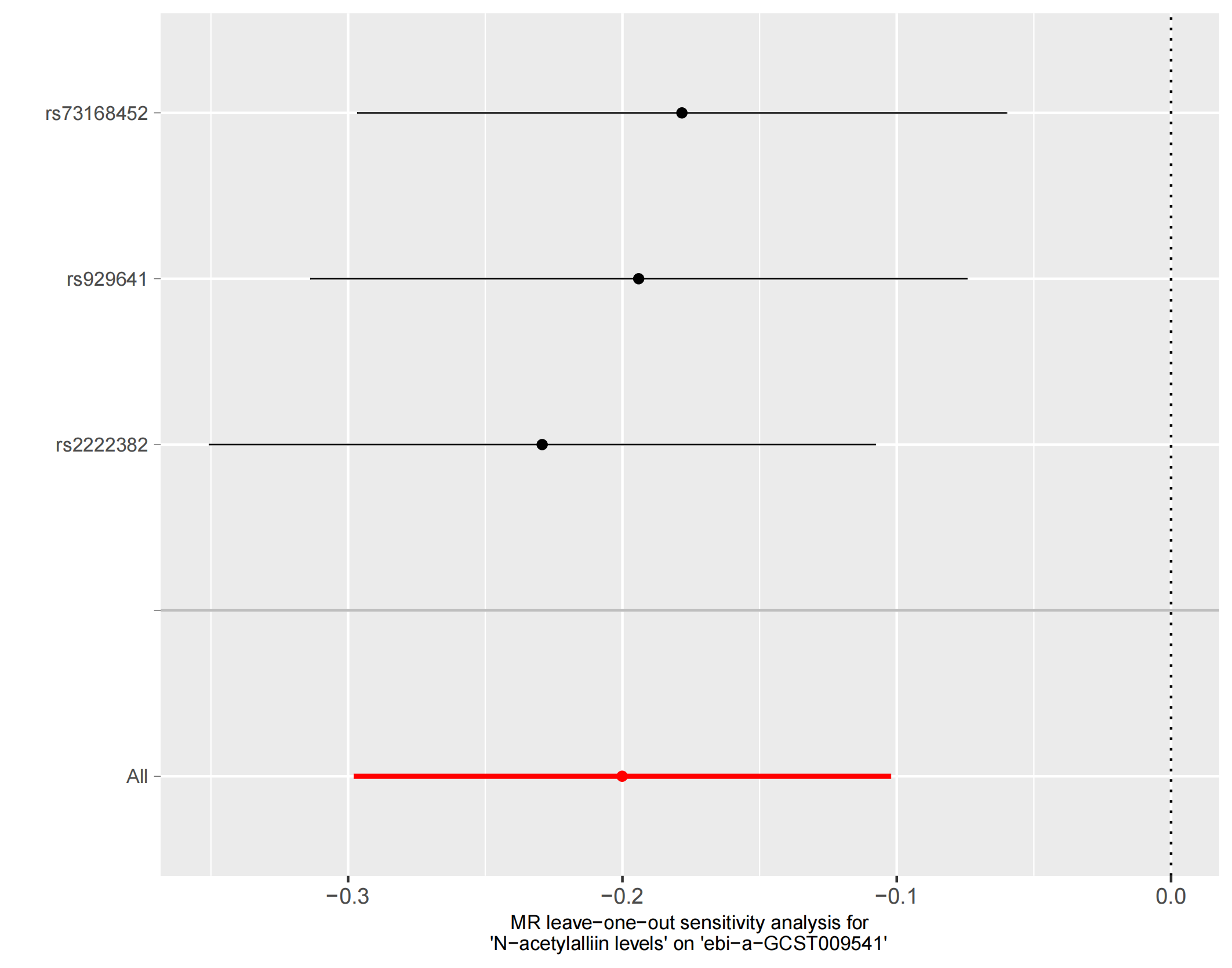 |
| --- | --- |
| 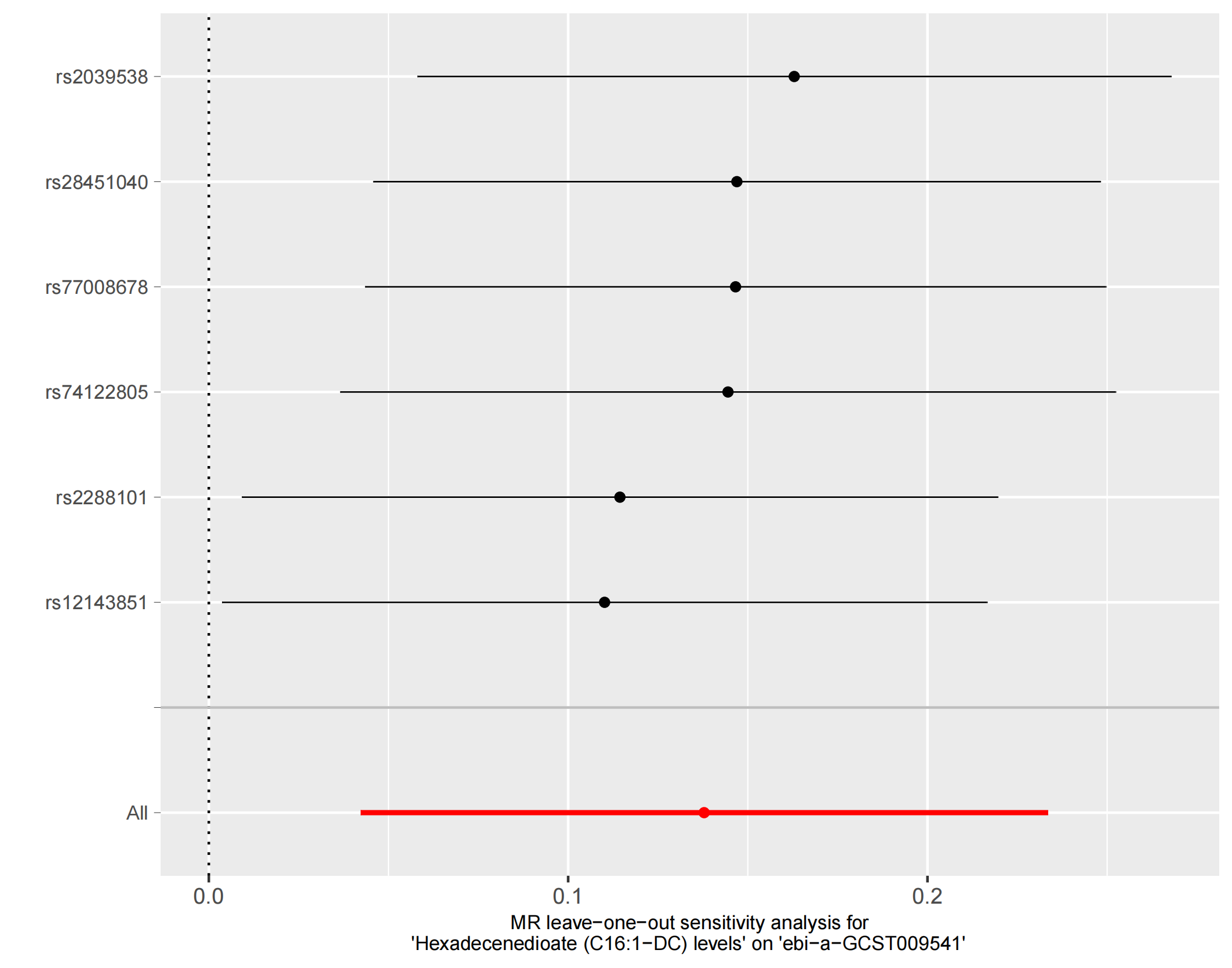 |  |

**Supplementary Figure S7.** The scatter plot in MR studies of Immune cells to Plasma Metabolites.

| 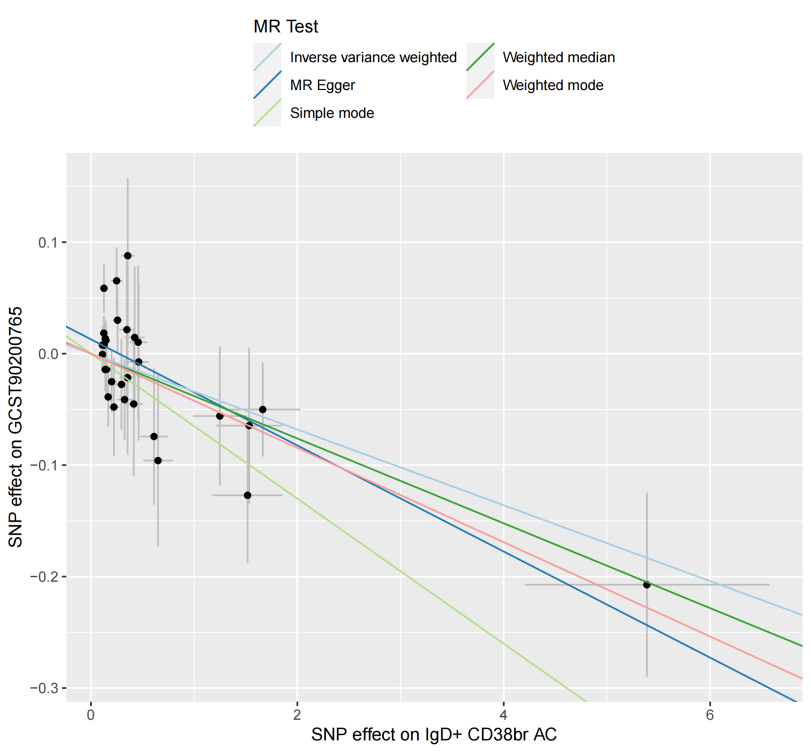 | 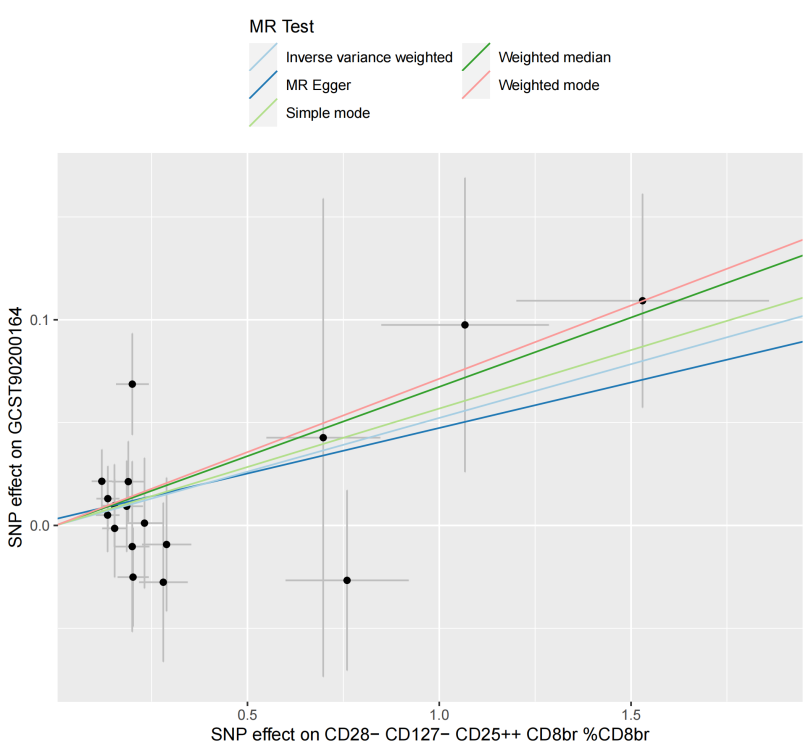 |
| --- | --- |
| 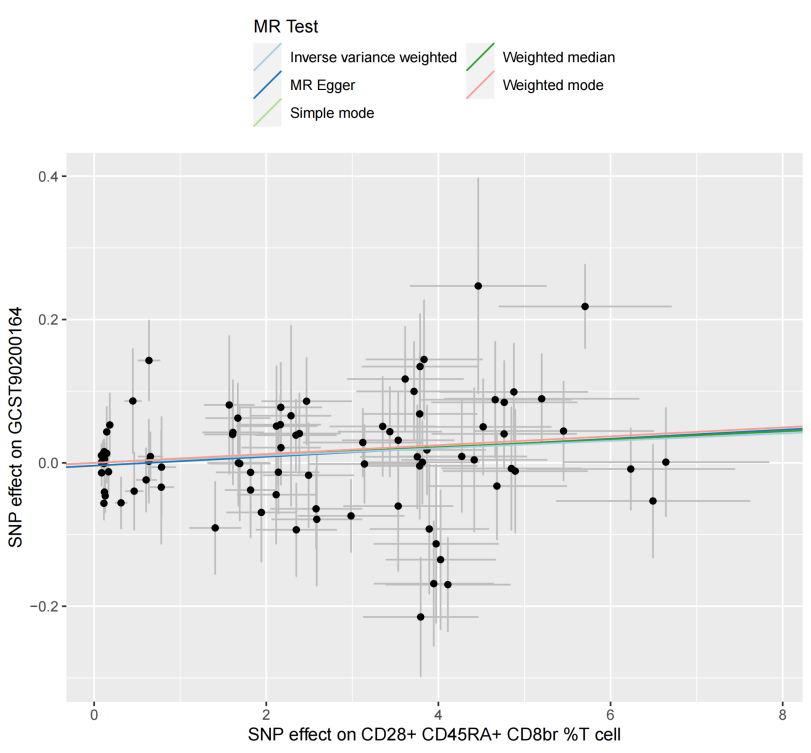 |  |

**Supplementary Figure S8.** The funnel plot in MR studies of Immune cells to Plasma Metabolites.

| 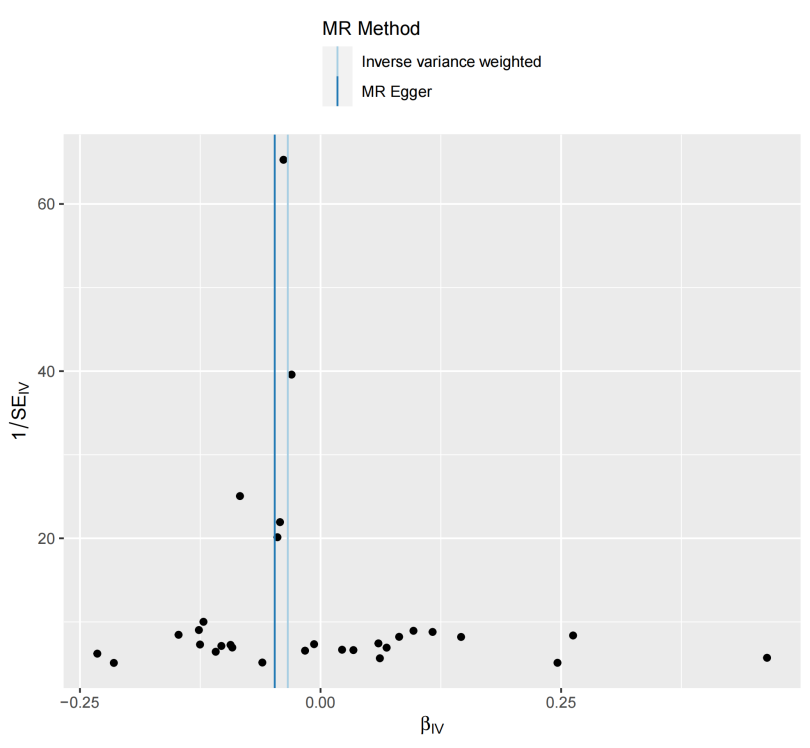 | 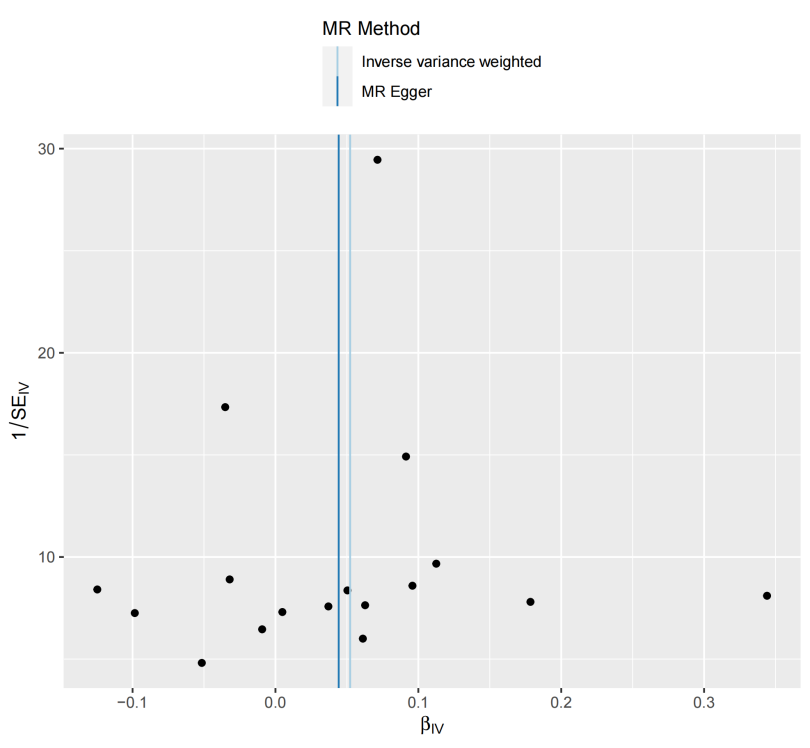 |
| --- | --- |
| 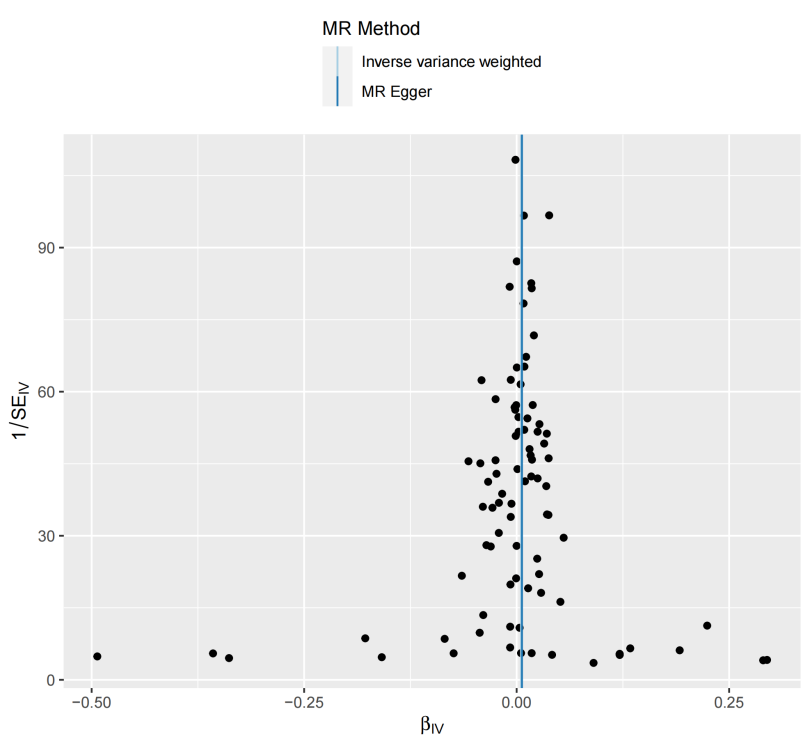 |  |

**Supplementary Figure S9.** The leave-one-out analysis results in MR studies of Immune cells to Plasma Metabolites.

| 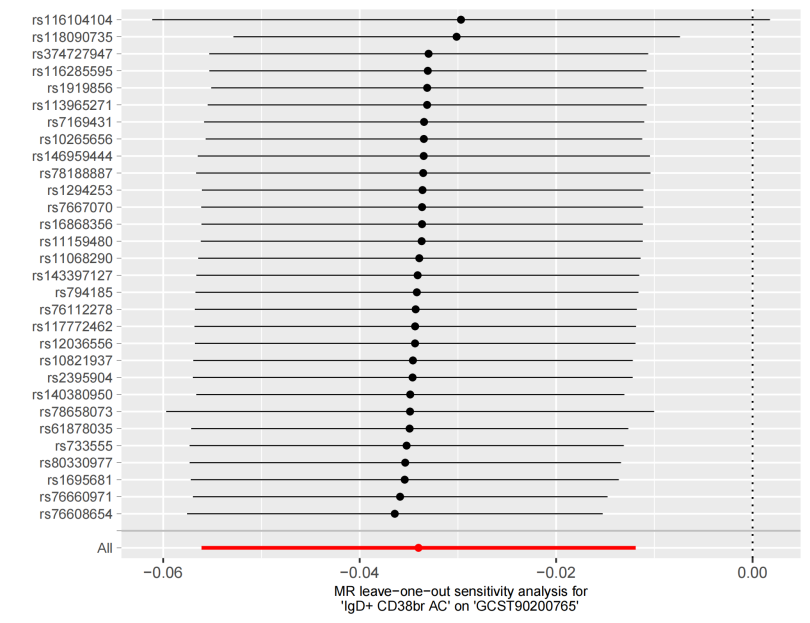 | 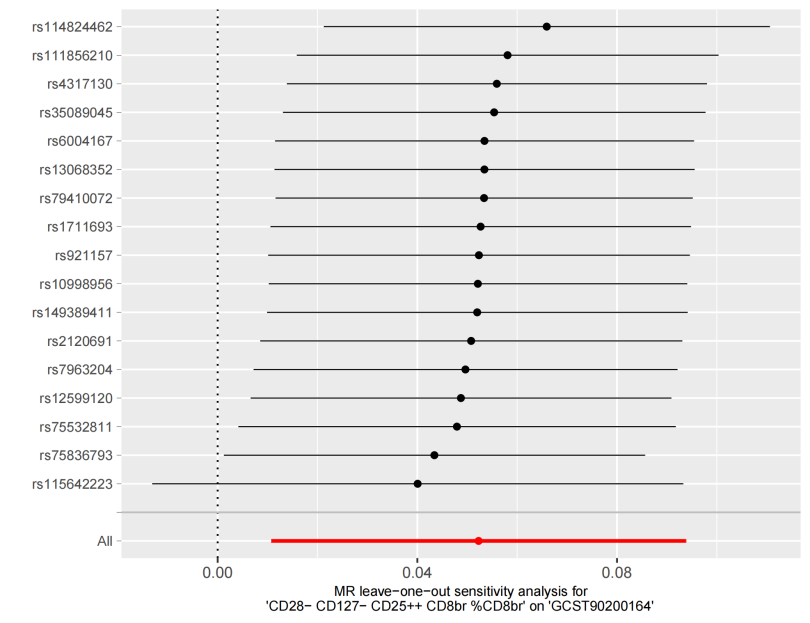 |
| --- | --- |
| 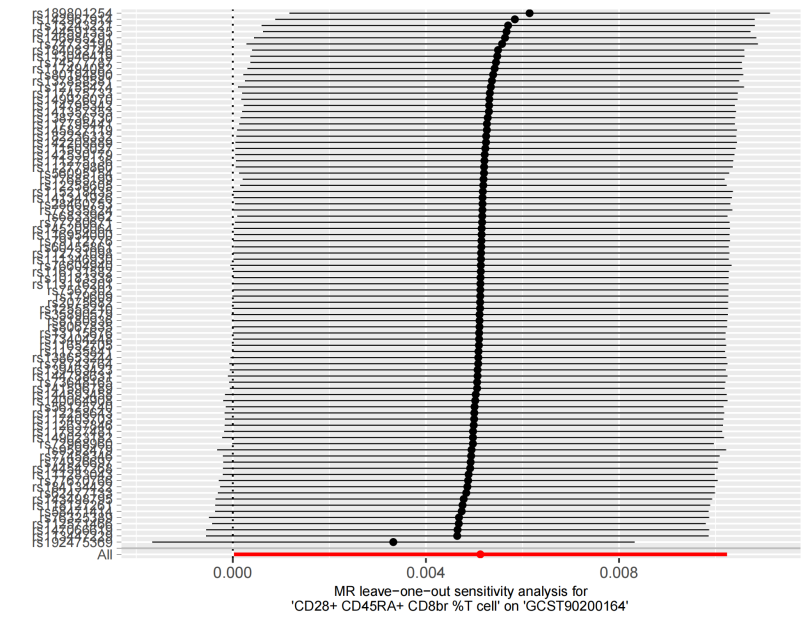 |  |
